# Mammary molecular portraits reveal lineage-specific features and progenitor cell vulnerabilities

**DOI:** 10.1083/jcb.201804042

**Published:** 2018-08-06

**Authors:** Alison E. Casey, Ankit Sinha, Rajat Singhania, Julie Livingstone, Paul Waterhouse, Pirashaanthy Tharmapalan, Jennifer Cruickshank, Mona Shehata, Erik Drysdale, Hui Fang, Hyeyeon Kim, Ruth Isserlin, Swneke Bailey, Tiago Medina, Genevieve Deblois, Yu-Jia Shiah, Dalia Barsyte-Lovejoy, Stefan Hofer, Gary Bader, Mathieu Lupien, Cheryl Arrowsmith, Stefan Knapp, Daniel De Carvalho, Hal Berman, Paul C. Boutros, Thomas Kislinger, Rama Khokha

**Affiliations:** 1 Princess Margaret Cancer Centre, Toronto, ON, Canada; 2 Informatics and Biocomputing Program, Ontario Institute for Cancer Research, Toronto, ON, Canada; 3 The Donnelly Centre, University of Toronto, Toronto, ON, Canada; 4 Structural Genomics Consortium, University of Toronto, Toronto, ON, Canada; 5 Nuffield Department of Clinical Medicine, Structural Genomics Consortium, University of Oxford, Oxford, UK; 6 Department of Medical Biophysics, University of Toronto, Toronto, ON, Canada; 7 Department of Pharmacology and Toxicology, University of Toronto, Toronto, ON, Canada; 8 Department of Laboratory Medicine and Pathobiology, University of Toronto, ON, Canada

## Abstract

The mammary epithelium depends on specific lineages and their stem and progenitor function to accommodate hormone-triggered physiological demands in the adult female. Perturbations of these lineages underpin breast cancer risk, yet our understanding of normal mammary cell composition is incomplete. Here, we build a multimodal resource for the adult gland through comprehensive profiling of primary cell epigenomes, transcriptomes, and proteomes. We define systems-level relationships between chromatin–DNA–RNA–protein states, identify lineage-specific DNA methylation of transcription factor binding sites, and pinpoint proteins underlying progesterone responsiveness. Comparative proteomics of estrogen and progesterone receptor–positive and –negative cell populations, extensive target validation, and drug testing lead to discovery of stem and progenitor cell vulnerabilities. Top epigenetic drugs exert cytostatic effects; prevent adult mammary cell expansion, clonogenicity, and mammopoiesis; and deplete stem cell frequency. Select drugs also abrogate human breast progenitor cell activity in normal and high-risk patient samples. This integrative computational and functional study provides fundamental insight into mammary lineage and stem cell biology.

## Abstract (precis)

Casey et al. integrate epigenomic, transcriptomic, and proteomic profiling of primary basal and luminal mammary cells to identify master epigenetic regulators of the mammary epithelium and uncover stem and progenitor cell vulnerabilities. They develop a pipeline to identify drugs that abrogate progenitor cell activity in normal and high-risk breast cancer patient samples in vitro and in vivo.

## Introduction

The mammary gland is a defining feature of mammals. Its study has provided new knowledge on organogenesis, differentiation programs, control of cell fate, and the molecular interplay that enables proliferation of tissue-specific progenitor cells (Hennighausen and Robinson, 2005). Elucidating the events that go awry in breast cancer formation requires a deep understanding of the normal adult breast. Recent discoveries of inherited single-nucleotide polymorphisms (Nguyen et al., 2015; Michailidou et al., 2017) that increase cancer risk will also benefit from information contextualizing their impact on the mammary epithelium.

The mammary epithelial hierarchy has two main lineages, basal and luminal, each of which contain progenitor cells. The luminal compartment comprises estrogen and progesterone receptor–positive (ER^+^PR^+^) and ER^−^PR^−^ cells. Lineage-tracing studies have demonstrated that under physiological conditions, basal, ER^+^PR^+^ luminal, and ER^−^PR^−^ luminal cells are each maintained by their own unipotent stem cells (Van Keymeulen et al., 2011, 2017; van Amerongen et al., 2012). A small number of mammary epithelial cells have been shown to reconstitute complete mammary structures when transplanted in vivo and have thus been termed mammary stem cells (Shackleton et al., 2006; Stingl et al., 2006; Eirew et al., 2008). However, whether bipotent adult stem cells contribute to the mammary epithelium in a physiological setting is controversial. Although some lineage-tracing studies have provided in situ evidence of bipotent stem cell activity (Rios et al., 2014; Wang et al., 2015), a subsequent statistics-based study has suggested that these results may result from a lack of labeling specificity (Wuidart et al., 2016), with questions remaining regarding both approaches (Rios et al., 2016).

Evidence suggests that stem and progenitor cells underlie cancer development and are cells of origin in aggressive breast cancer subtypes. Luminal progenitors are expanded in BRCA1 mutation carriers and linked to basal-like breast cancers, whereas stem- and progenitor-enriched basal cells are associated with claudin-low breast cancers (Lim et al., 2009; Molyneux et al., 2010; Shehata et al., 2012). Cancer risk has also been correlated to the number of stem cell divisions inherent to tissue homeostasis (Tomasetti et al., 2017); this concept is relevant to the breast, which undergoes extensive tissue remodeling during the female lifespan in response to hormones. Molecular interventions centered on targeting stem and progenitor cells thus offer promising strategies for breast cancer chemoprevention.

Mammary stem and progenitor cells typically show undetectable expression of ER and PR yet expand during the progesterone-high phase of the reproductive cycle and pregnancy to drive sex hormone–induced mammopoiesis. Effects of circulating progesterone on ER^−^PR^−^ stem and progenitor cells are mediated via paracrine factors secreted by ER^+^PR^+^ luminal cells (Asselin-Labat et al., 2010; Joshi et al., 2010, 2015a; Shiah et al., 2015). Multiple lines of evidence support that progesterone exposure elevates breast cancer risk. In mice, mammary tumorigenesis is lower after PR deletion or treatment with a PR antagonist (Lydon et al., 1999; Sigl et al., 2016). Early menarche or late menopause is a known risk factor in breast cancer (Kelsey et al., 1993), and oophorectomy is protective in high-risk women (Kauff et al., 2002; Eisen et al., 2005; Kotsopoulos et al., 2016). Population studies show that breast cancer risk is higher for women on hormone replacement therapy formulations containing progestins (Chlebowski et al., 2015; Joshi et al., 2015b,c), and high serum progesterone and RANKL correlate with increased risk in postmenopausal women without genetic predisposition (Kiechl et al., 2017). Conversely, progestins exert antiproliferative effects on ER^+^PR^+^ breast cancer cells (Mohammed et al., 2015). Because ER^−^PR^−^ and ER^+^PR^+^ mammary cells exhibit divergent responses to progesterone, it is critical to understand the molecular circuitry underlying sex hormone responsiveness.

To date, profiling of primary mammary subsets has focused on transcriptome and/or epigenome analyses (Kendrick et al., 2008; Lim et al., 2010; Maruyama et al., 2011; Gascard et al., 2015; Pellacani et al., 2016), with few studies done in controlled hormone states (Pal et al., 2013; Dos Santos et al., 2015; Shiah et al., 2015). Yet studies have not defined the open chromatin landscapes or proteomes of the basal and luminal lineages, nor have they integrated successive levels of gene regulation. Here, we constructed chromatin–DNA–RNA–protein mammary molecular portraits, which include newly generated and matched methylome, assay for transposase-accessible chromatin using sequencing (ATAC-seq), and proteome data. Proteomics was then extended to three cell subsets from contrasting progesterone states: ER^−^PR^−^ basal, ER^−^PR^−^ luminal progenitor, and ER^+^PR^+^ luminal cells. This global mammary resource exposes statistical relationships across four successive levels of regulation, yielding new insights into the DNA and chromatin states of key transcription factor binding sites (TFBSs) and highlighting distinct expression patterns of lineage-restricted versus total genes. Finally, mammary portraits uncover new pathways controlling stem and progenitor cell function and drugs that exert cytostatic effects to limit sex hormone–driven adult stem and progenitor expansion and mammopoiesis. Drugs also impede human breast cell clonogenicity in specimens from normal and high-risk women.

## Results

### Union of basal and luminal mammary epigenomes, transcriptomes, and proteomes

We set out to comprehend the two mammary lineages, which are each enriched for distinct stem and progenitor cells, by quantifying global relationships between basal and luminal cell epigenomes, transcriptomes, and proteomes in adult mice. To normalize sex hormone exposure, mice were ovariectomized and treated with 17β-estradiol and progesterone (designated EP); primary basal (CD24^lo-med^CD49f^hi^) and luminal (CD24^hi^CD49f^lo^) cells were FACS-purified. We performed reduced representation bisulfite sequencing (RRBS) and ATAC-seq to identify DNA methylation and open chromatin regions, respectively (Meissner et al., 2005; Buenrostro et al., 2013). For transcriptomes, we leveraged our published RNA abundance data (Shiah et al., 2015). For proteomes, we performed ultra-high pressure liquid chromatography/mass spectrometry (UPLC-MS) and identified 4,213 proteins.

**Figure 1.**
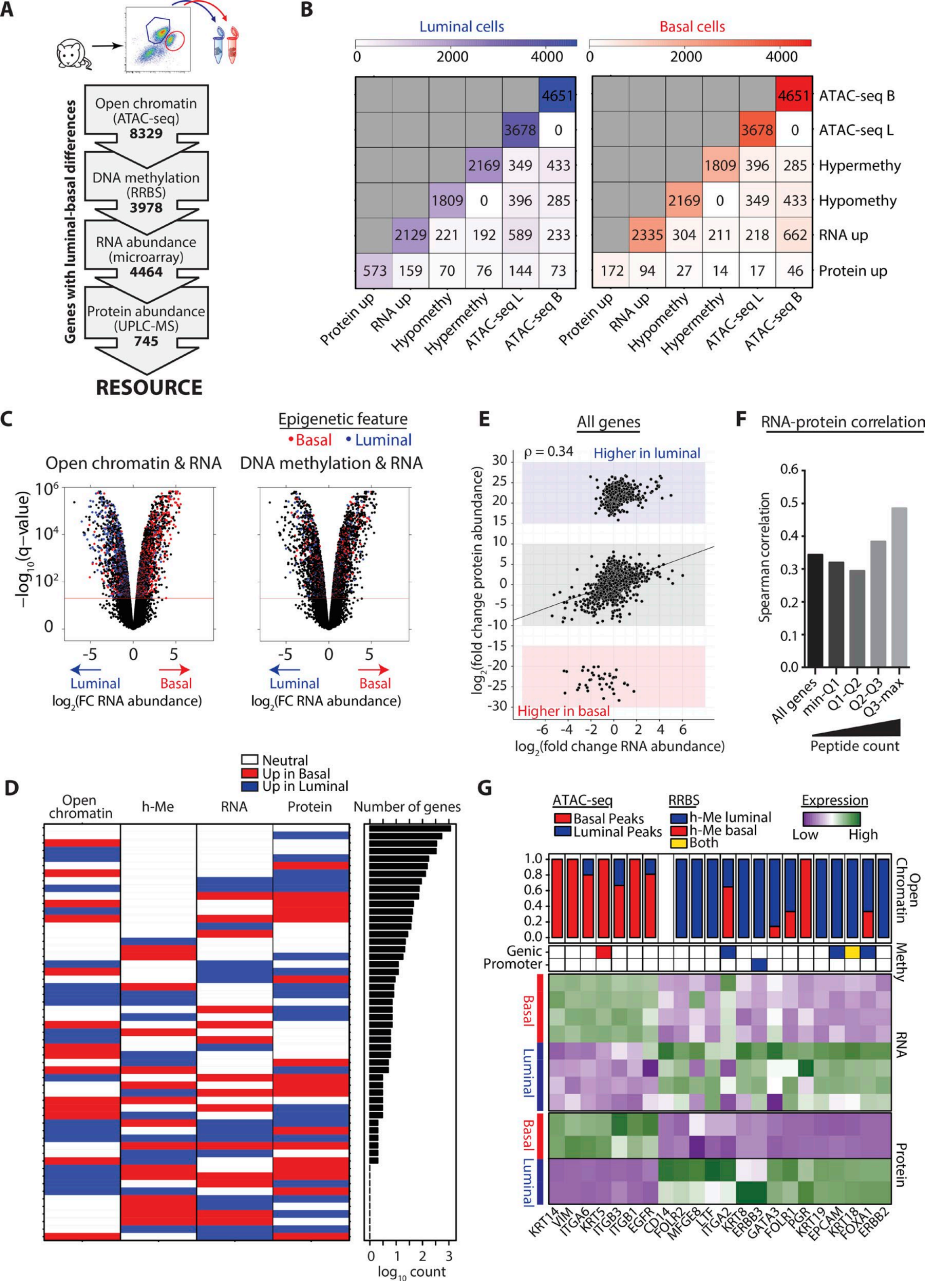
**Integrated proteomic, transcriptomic and epigenomic profiling of basal and luminal mammary cells. (A)** Schematic depicts analyses performed on EP-treated basal and luminal cells. Biological replicates: ATAC-seq, RRBS, and UPLC-MS, *n* = 2; microarray, *n* = 4. **(B)** Tables show numbers of genes associated with protein or RNA up-regulation, DNA hypo- or hypermethylation, and lineage-restricted ATAC-seq peaks, in basal (B) and luminal (L) cells. **(C)** Volcano plots show log_2_(fold change RNA abundance) across mammary cell compartments; color coding shows genes associated with ATAC-seq peaks or DNA hypomethylation specific to basal/luminal cells. **(D)** Heatmap depicts genes classified based on their relationship states between open chromatin, DNA hypomethylation (h-Me), RNA, and protein abundances. Bar plot shows the number of genes in each state on a log_10_ scale. **(E)** Graph shows log_2_(fold change) of RNA versus protein abundance of all genes found in both microarray and UPLC-MS datasets. **(F)** Graph shows Spearman’s correlation (ρ) of log_2_(fold change) in RNA versus protein abundance. Genes were divided into quantiles (Q1–Q4) based on their peptide counts (biological replicates: UPLC-MS, *n* = 2; microarray, *n* = 4). **(G)** Heatmaps show z-scores of protein and RNA abundance of known marker proteins in basal and luminal subsets. Color coding indicates gene hypomethylation, and bar chart shows the relative proportion of total ATAC-seq peaks detected in basal or luminal cells.

Global integration of data revealed a positive relationship of open chromatin regions with both RNA and protein abundance (Fig. 1, A–C). Specifically, genes with higher abundance in basal or luminal cells associated more with ATAC-seq peaks detected in that, versus the other, cell type (e.g., for RNA basal, 662/880 or 75%; for luminal, 589/822 or 72%). Minimal relationship existed between DNA hypomethylation and RNA or protein abundance (e.g., for RNA basal, 304/515 or 59%; for luminal, 221/413 or 54%); DNA methylation at promoter regions again showed no relationship with RNA abundance (Fig. S1 A). Genomic regions enrichment of annotations tool (GREAT) was used to explore functional significance of lineage restricted open chromatin regions (McLean et al., 2010), as shown in Fig. S1 B and Table S1. ErbB-2 class receptor binding was enriched in basal cells (*q* = 0.004), and numerous pathways critical to cell differentiation, survival and breast cancer were enriched in luminal including c-KIT, NOTCH, and GH receptor signaling (Fig. S1 B and Table S1). Next, we classified genes based on their chromatin–DNA–RNA–protein relationships, quantifying the frequency and probability of specific states (Fig. 1 D and Table S2 for log-odds ratios and p-values). One-third of genes identified in all four datasets did not differ between basal and luminal cells (1,147/3,424) and mostly represented common cellular processes (Fig. S1 C). Open chromatin regions statistically associated with increased RNA abundance in both mammary lineages (p-values: basal = 1.51 × 10^−9^, luminal = 1.45 × 10^−7^; Table S2). In basal cells, open chromatin also associated with increased protein abundance (P = 4.52 × 10^−7^; Table S2); luminal cell relationships were more complex, with ATAC-seq peaks linked to both increased (P = 5.63 × 10^−6^; Table S2) and decreased (P = 0.022; Table S2) protein abundance.

It has been shown that RNA abundances only weakly correlate with protein levels (Kislinger et al., 2006; Cox et al., 2009; Rugg-Gunn et al., 2012). We observed a positive association between increased RNA and protein abundance (p-values: basal = 1.86 × 10^−48^; luminal = 1.36 × 10^−31^; Table S2). RNA–protein correlations were higher for more abundant proteins (Spearman’s ρ = 0.49), although correlation at the global level was weak (Spearman’s ρ = 0.34; Fig. 1, E and F). Overall, 35% of genes changed at the RNA or protein level across mammary lineages (basal 356/3,424; luminal 835/3,424); of these, only 3% displayed the conventional pattern of more open chromatin and increased RNA and protein (basal 38/3,424; luminal 76/3,424). We next interrogated a 22-basal-luminal marker gene signature for their ATAC-seq, DNA methylation, RNA, and protein status (Fig. 1 G). Here, increased lineage restricted open chromatin associated with higher gene expression (20/22; 91%); most genes were not DNA methylated (16/22; 73%); and 21/22 exhibited concordant RNA and protein abundance. Altogether, although tightly controlled marker genes exhibit open chromatin and concordant RNA/protein expression, most others fall outside of this conventional regulation pattern. This integrative computational analysis provides insight into relationships between chromatin structure and translational/posttranslational control.

### Key TFBSs are differentially methylated in basal versus luminal cells

**Figure 2.**
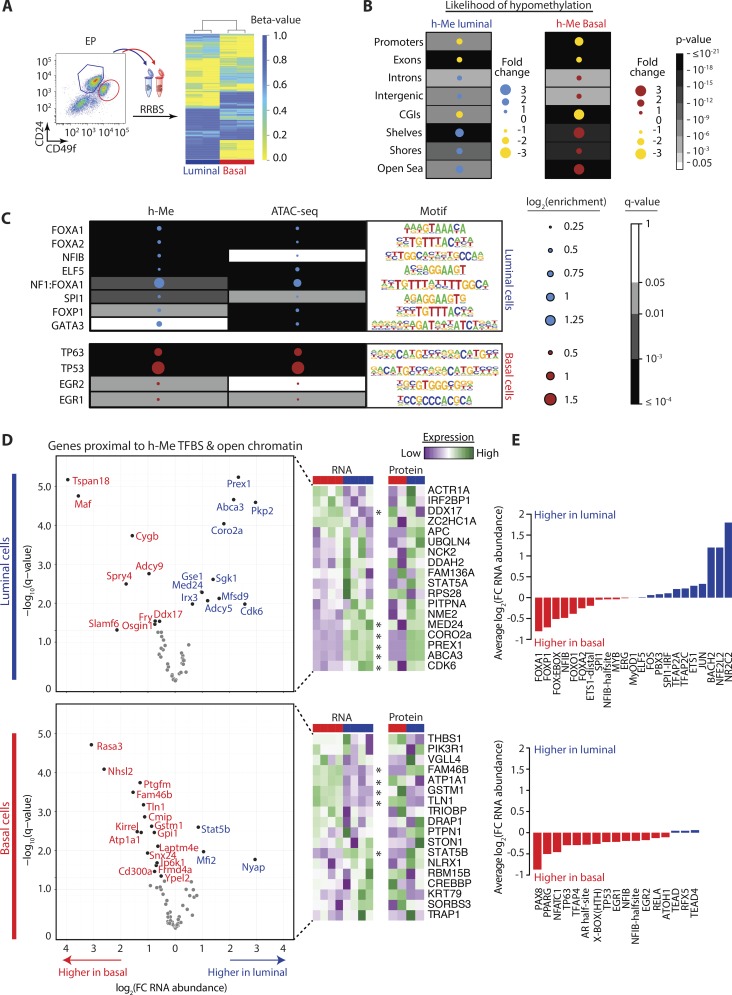
**TFBSs are hypomethylated in basal and luminal cells. (A)** RRBS of basal and luminal cells from EP-treated mice (biological replicates, *n* = 2). Heatmap shows unsupervised hierarchical clustering and β-values of luminal and basal subsets. **(B)** Dot maps show likelihood of DNA hypomethylation (h-Me) occurring at specific gene locations or in different types of methylation regions. **(C)** Dot maps show log_2_(enrichment over background) and *q*-values for TFBS that are hypomethylated and/or enriched in open chromatin regions in basal or luminal cells. Tables show HOMER motif logos. **(D)** Volcano plots show differences in RNA abundance for genes located near h-Me TFBS and open chromatin, in basal and luminal cells. Heatmaps show RNA and protein abundance of proximal genes identified in both transcriptomic and proteomic datasets; asterisks mark genes with significant differences in RNA abundance across mammary lineages. **(E)** Bar charts show mean log_2_(fold change) in RNA abundance for all genes proximal to the indicated h-Me TFBS, in basal or luminal cells.

Overall, less DNA methylation exists in the basal compartment, with more differentially methylated cytosines (DMCs) hypomethylated in basal (5,168) versus luminal (4,095) cells. Methylomes clustered separately (Fig. 2 A) with DMCs more likely to occur at introns or intergenic sites and in shelf, shore, or open-sea regions (Fig. 2 B). Because our multimodal data suggests that DNA methylation does not regulate proximal gene expression, we reasoned it may influence cell state by controlling transcription factor binding. TFBS analysis revealed several motifs hypomethylated in basal versus luminal cells, with many also enriched in lineage-restricted functional open chromatin (Figs. 2 C and S1 D). These included TFBSs for key transcription factors such as FOXA1, ELF5, GATA3, and TP63, which are essential regulators of mammary morphogenesis, cell fate, differentiation, and lineage identity. Other TFBSs associated with DNA hypomethylation and open chromatin regions belonged to TP53 and EGR1 in basal and FOXA2, SPI1, and FOXP1 in luminal cells.

We used our expression data to interrogate RNA and protein abundance of protein-coding genes proximal to hypomethylated TFBSs and open chromatin regions (≤250 bp, lineage-restricted or shared; Fig. 2 D and Tables S3 and S4). Most proximal genes did not exhibit significant changes in their RNA abundance (basal 42/61, 69%; luminal 37/57, 65%; Tables S3 and S4). This may signify the inherent complexity of epigenetic control over gene expression or that hypomethylated TFBSs are located in enhancer regions. In analogous human breast epithelial subsets, these same TFBSs associate with cell type–specific enhancers shown via H3K4me1, H3K4me3, and H3K27ac chromatin immunoprecipitation sequencing (ChIP-seq; Pellacani et al., 2016), with methylome analysis highlighting a strong overlap between hypomethylated genome regions and enhancer chromatin states (Gascard et al., 2015).

For the proximal genes that did change with hypomethylation, most significant differences in RNA were reflected at the protein level. One exception was FAM46B, with a clear discordance in RNA/protein abundance (Fig. 2 D and Table S3). For instance, 16 of 19 genes in basal cells had elevated expression (Fig. 2 D and Table S3) and represented known (*Tln1*; Deugnier et al., 2002) or novel (e.g., *Gstm1* and *Atp1a1*) basal cell features. In luminal cells, altered genes both increased (11/20; 55%) and decreased (9/20; 45%; Fig. 2 D and Table S4) in expression. Among the up-regulated luminal genes are *Prex1*, *Abca3*, and *Cdk6*: *Prex1* is overexpressed in ER^+^ and HER2^+^ breast cancers (Marotti et al., 2017), whereas *Abca3* and *Cdk6* are higher in normal human luminal cells (Lucas et al., 2004; Schimanski et al., 2010); *Abca3* is an ERα target gene; its loss is an adverse risk factor for breast cancer recurrence and promotes acquisition of mesenchymal-like characteristics in lung epithelial cells (Lin et al., 2004; Kaltenborn et al., 2012). Deletion of the CDK4/6 inhibitor p18^INK4C^ stimulates luminal progenitor expansion and mammary tumor formation in mice (Pei et al., 2009).

Interestingly, genes tended to be located near ≥2 hypomethylated TFBSs in both mammary lineages (basal 44/61 or 72%; luminal 45/59 or 76%; Tables S3 and S4). To probe effects of different hypomethylation events on gene expression, we calculated the mean RNA fold-change associated with each TFBS (Fig. 2 E). In basal cells, most TFBSs associated with increased RNA abundance (Fig. 2 E). In luminal cells, motifs belonging to NR2C2, NFE2L2, and BACH2 associated with strong increases in RNA, whereas FOXA1, FOXP1, and NFIB associated with decreased RNA (Fig. 2 E). These integrated analytics of primary mammary cells challenge the classic view that DNA methylation, whether in promoter regions or otherwise, regulates nearest gene expression. Rather, our findings suggest a major role for DNA methylation is controlling TFBS accessibility on the genome.

### Defining the protein landscapes of mammary cell lineages

**Figure 3.**
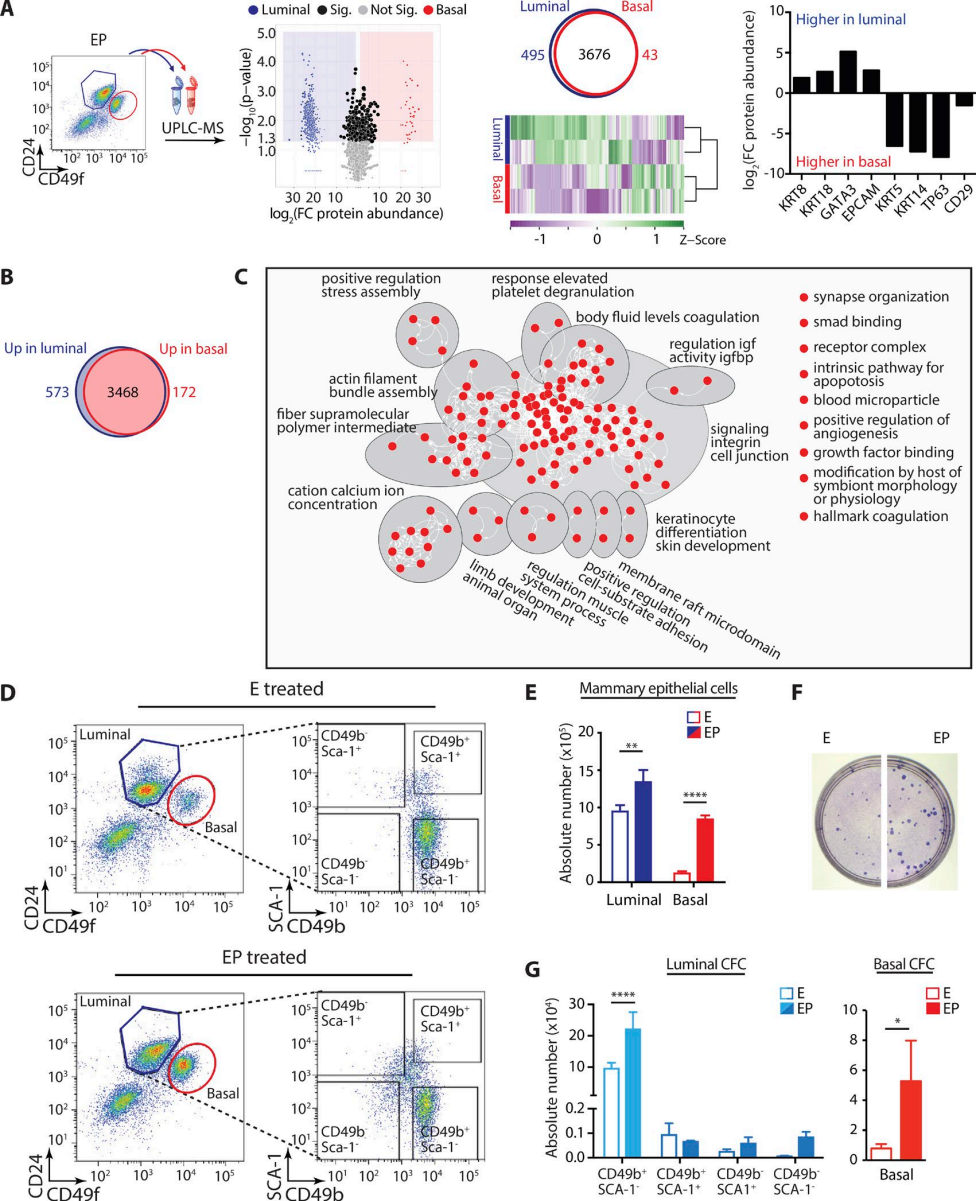
**Progesterone stimulates expansion of basal and luminal progenitor cells. (A)** UPLC-MS of basal and luminal cells from EP-treated mice (biological replicates, *n* = 2). Volcano plot shows differential protein expression in mammary cell compartments. Proteins detected in only one compartment are shown in blue (luminal) or red (basal); proteins detected in both lineages and altered across compartments are shown in black (fold-change ≥2, P ≤ 0.05). Venn diagram depicts number of identified proteins per cell compartment. Heatmap shows unsupervised hierarchical clustering and z-scores of protein abundance across basal and luminal subsets. Bar chart shows log_2_(fold change) in luminal and basal marker protein abundance. **(B)** Venn diagram depicts number of proteins differentially expressed across the basal and luminal mammary lineages (≥2-fold change, P < 0.05). **(C)** Enrichment map summarizes results of GSEA pathway analysis for proteins up-regulated in basal compared with luminal cells (FDR ≤0.05). Up-regulated pathways include regulation of insulin-like growth factor (IGF) activity by insulin-like growth factor binding protein (IGFBP). Nodes represent biological pathways that were automatically annotated and organized into themes using Cytoscape; biological themes are labeled and depicted via gray ellipses. **(D)** Left: Flow cytometry analysis of luminal (CD24^+^CD49f^lo^) and basal (CD24^−^CD49f^hi^) primary mammary cells, purified from three pairs of glands (second, third, and fourth) of E- or EP-treated mice. Right: The luminal subset further subdivided using the CD49b and SCA-1 cell-surface markers (Shehata et al., 2012). **(E)** Bar chart shows absolute number of basal and luminal cells from E- or EP-treated mice; biological replicates, *n* = 3; error bars represent SD. **(F)** Photographs of representative CD49b^+^SCA-1^-^ luminal CFC plate from E- versus EP-treated mice. **(G)** Bar charts show absolute number of CFC within the different luminal or basal subsets, in E- or EP-treated mice (*n* = 3, error bars represent SD). **(E and G)** Statistical significance was calculated using two-tailed *t* test (basal CFC; *, P < 0.05) or two-way ANOVA and Sidak’s multiple comparisons test (mammary epithelial cells, luminal CFC). Multiple comparisons testing was performed with a 0.05 significance level and 95% confidence interval. Statistically significant differences are indicated by asterisks, which denote size of significance levels. **, P ≤ 0.01; ****, P < 0.0001.

We next interrogated mammary cell proteomes that exhibited separate clustering and hallmark characteristics of the basal (KRT5, KRT14, CD29, TP63) and luminal (KRT8, KRT18, EPCAM, GATA3) compartments (Fig. 3 A and Table S5). Some proteins were detected in only one lineage, resulting in absolute log_2_(fold change) >15 (Fig. 3 A and Table S5). Comparison of basal versus luminal proteomes revealed 745 differentially expressed proteins (Fig. 3 B; P < 0.05, fold change ≥2). More proteins were up-regulated in luminal cells (573 vs. 172) likely because of greater heterogeneity within this compartment. Intriguingly, gene set enrichment analysis (GSEA) demonstrated that luminal up-regulated proteins lacked enrichment for any biological terms, whereas those up-regulated in basal cells were enriched for 139 different functions or pathways (false discovery rate [FDR] ≤0.05; Table S6) that clustered into 13 groups (Fig. 3 C), reflecting novel or known features of basal/myoepithelial cells (Deugnier et al., 2002). Further to enabling chromatin–DNA–RNA–protein comparisons and providing a biological baseline for future queries, our mammary proteomes show the feasibility of performing label-free MS-based shotgun proteomics on small numbers of FACS-sorted, primary cells (∼100,000 cells).

### Mammary cell proteomes and their hormone responsiveness

We next set out to define proteomes of stem- and progenitor-enriched mammary populations. For this, equal numbers of FACS-purified ER^−^PR^−^ basal, ER^−^PR^−^ luminal progenitor, and ER^+^PR^+^ luminal cells were pooled from ovariectomized mice treated with 17β-estradiol alone (E) or 17β-estradiol and progesterone (EP). These treatments mimic contrasting phases of the natural estrous cycle where addition of progesterone induces robust stem and progenitor expansion and changes at the transcriptomic level over estrogen alone (Shiah et al., 2015). Flow cytometry and matched colony-forming capacity (CFC) assays confirmed that EP increased absolute numbers of basal and luminal cells (Fig. 3, D and E) as well as progenitors (Fig. 3, F and G). As expected, most luminal progenitors resided in the CD49b^+^Sca-1^−^ population (Fig. 3 G; Shehata et al., 2012).

**Figure 4.**
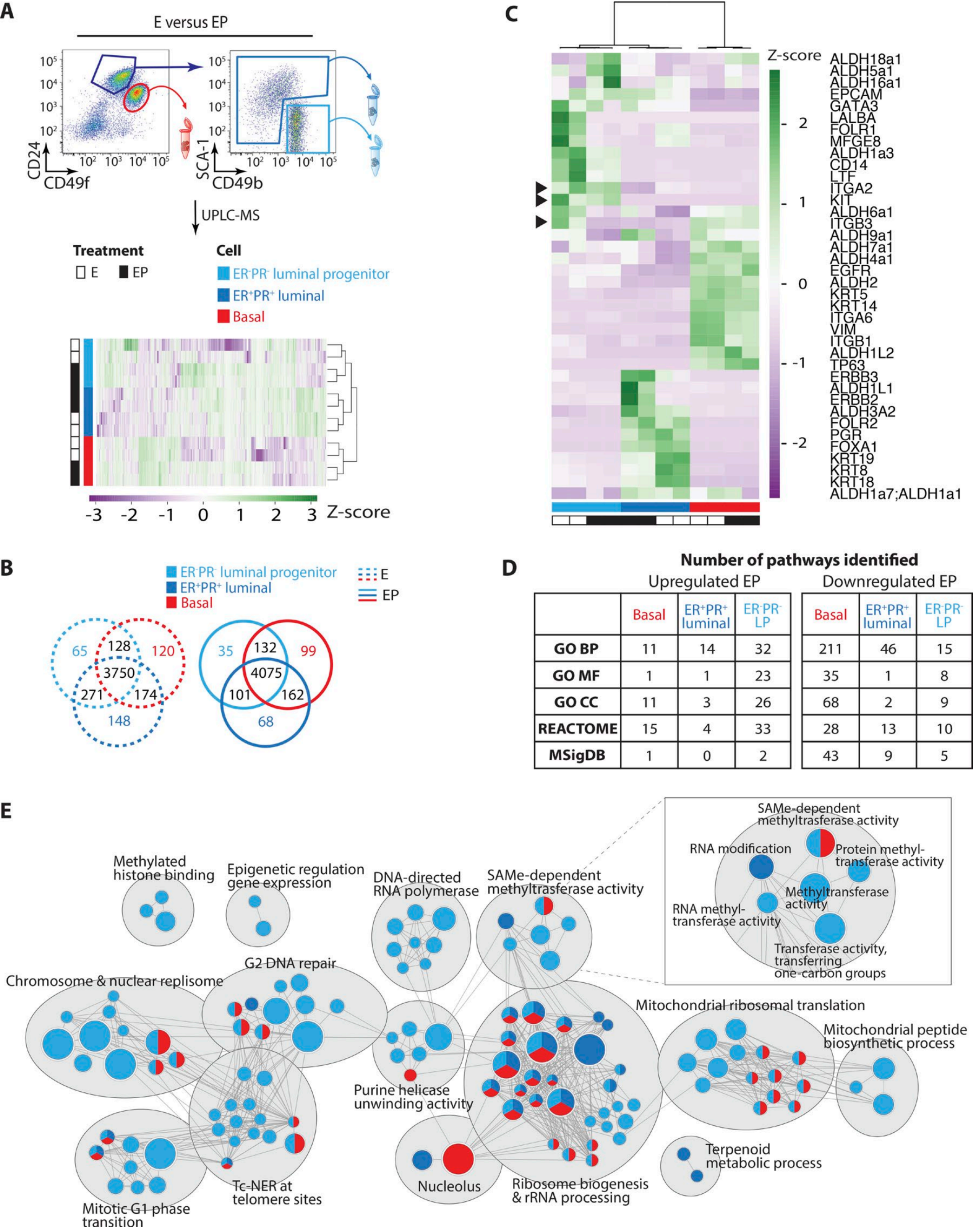
**Defining the protein composition and hormone responsiveness of mammary epithelial subsets. (A)** UPLC-MS of ER^−^PR^−^ basal, ER^−^PR^−^ luminal progenitor, and ER^+^PR^+^ luminal cells from E- and EP-treated mice (biological replicates, *n* = 2). Heatmap shows unsupervised hierarchical clustering and z-scores of protein expression across samples. **(B)** Venn diagrams depict number of proteins identified in ER^−^PR^−^ basal, ER^−^PR^−^ luminal progenitor, or ER^+^PR^+^ luminal cells after E or EP treatment. **(C)** Heatmap shows unsupervised hierarchical clustering and marker protein expression across cell compartments and hormone states. Arrowheads denote ITGA2/CD49b, c-KIT, and ITGB3/CD61 luminal progenitor marker proteins. **(D)** Tables summarize GSEA results, detailing the numbers and types of gene sets enriched for proteins up- or down-regulated by progesterone in each mammary subpopulation (FDR ≤0.05). **(E)** Enrichment map visualizes results of GSEA for proteins up-regulated in EP compared with E proteomes. Nodes represent biological pathways that were automatically annotated and organized into themes using Cytoscape; biological themes are labeled and depicted via gray ellipses. Colors of nodes show which cell types were enriched for specific pathways (FDR ≤0.05), with multicolored nodes depicting pathways up-regulated by progesterone in two or more subpopulations: ER^−^PR^−^ basal (red), ER^−^PR^−^ luminal progenitor (light blue), and ER^+^PR^+^ luminal (darker blue) cells. Node size is proportional to the number of associated genes.

In total, proteomes identified 4,672 proteins that clustered first within cell fractions and then hormone states (Fig. 4, A and B; and Table S7). We observed a strong overlap between the well-known luminal progenitor markers ITGA2/CD49b, ITGB3/CD61 and c-KIT (Fig. 4 C, arrowheads) only after progesterone inclusion. Specifically, sex hormones induced a marked decline in ITGB3/CD61 that was not mirrored by either ITGA2/CD49b or c-KIT. This was independently verified by flow cytometry, which showed no difference between CD49b^+^Sca-1^−^ or c-KIT^+^ luminal progenitor numbers after EP treatment, but significantly fewer ITGB3/CD61^+^ luminal cells (Fig. S2 A). These data indicate that progesterone-driven loss of luminal progenitors is limited to the ITGB3/CD61^+^ subset and care should be taken when using this marker as an indicator of overall luminal progenitor activity, especially in the context of sex hormones. ALDH enzymatic activity also marks luminal progenitor cells and is detected via commercially available kits; however, the specific ALDH isoforms responsible for this remain unclear. We found pronounced ALDH heterogeneity across mammary cell compartments, with ALDH1a3, ALDH5a1, ALDH6a1, ALDH16a1, and ALDH18a1 being higher in luminal progenitors, yet many other isoforms exhibit selectivity for basal or ER^+^PR^+^ luminal cells (Fig. 4 C). Proteomics thus afforded new insight into mammary marker proteins.

We combined discovery proteomics with GSEA and enrichment map analyses to illustrate protein changes driven by progesterone in each mammary subpopulation (Fig. 4, D and E; Fig. S2, B and C; and Tables S8 and S9). More pathways were up-regulated in ER^−^PR^−^ luminal progenitors than in either of the other two cell compartments (Fig. 4, D and E; and Table S8). Despite marked differences existing across mammary lineages, we noted high overlap of progesterone-stimulated pathways in ER^−^PR^−^ luminal progenitor and basal cells; these were associated with nuclear changes, cell replication, and cell metabolism (Fig. 4 E). Progesterone drove terms linked to epigenetic processes primarily in ER^−^PR^−−^ luminal progenitor cells, with *S*-adenosylmethionine (SAMe)–dependent methyltransferase activity also up-regulated in the basal compartment (Fig. 4 E). Protein abundances associated with SAMe-dependent methyltransferase activity are shown in Fig. S2 B. SAMe is the primary methyl donor for methyltransferase enzymes and is thus intrinsic to chromatin remodeling and epigenetic modifications (Loenen, 2006). Fewer pathways were up-regulated by progesterone in ER^+^PR^+^ luminal cells (Fig. 4 E). Strikingly, a large number of pathways are down-regulated by EP in basal cells (Fig. S2 C and Table S9). Altogether, proteomics uncovered new focal points and distinctions in mammary cell molecular makeup, highlighting epigenetics as a putative mechanism for dictating lineage identity and hormone response.

### Progesterone up-regulates epigenetic master regulators in the mammary epithelium

ER^−^PR^−^ mammary cell populations exhibit a mitogenic response to progesterone and contain likely cells of origin for aggressive breast cancers. We leveraged the concept that epigenetic pathways underscore adult stem and progenitor cell expansion to identify vulnerabilities of these primitive cell types. Epigenetic targets are a highly active area of drug discovery, as evident by the recent development of many high-quality and specific chemical probes (Huston et al., 2015). We rationalized that drugs, if matched to key epigenetic regulatory proteins within the basal or luminal compartments, can serve as a means to create cytostatic effects and deplete stem- and progenitor-enriched populations.

**Figure 5.**
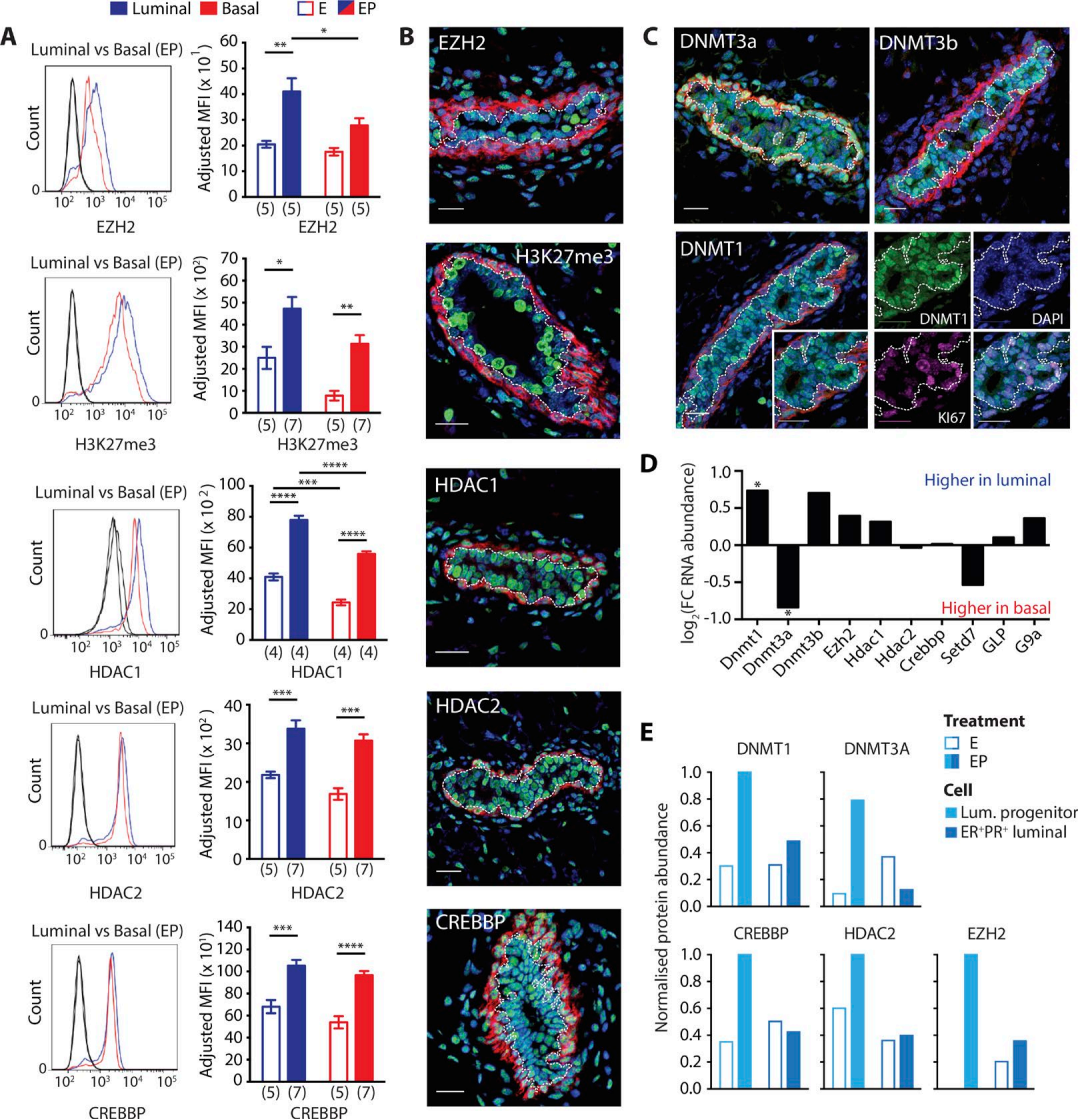
**Lineage specificity and hormone responsiveness of epigenetic master regulators. (A)** Intracellular flow cytometry of epigenetic targets in mammary epithelial cells. Example histograms show intensity staining for proteins compared with isotype Fc controls (black) in basal (red) and luminal (blue) cells on a log scale. Bar charts show adjusted MFI for each mammary population. Number of biological replicates is shown in brackets under graphs. Error bars represent SEM. Statistical significance was calculated using two-way ANOVA and Tukey’s multiple comparisons test performed with a 0.05 significance level and 95% confidence interval. Statistically significant differences are indicated by asterisks, which denote size of significance levels. **(B and C)** IF staining of mammary ductal structures in EP-treated mice: DAPI (blue), Ki67 (magenta), basal lineage marker KRT14 (red), and indicated epigenetic marks or proteins (green). Luminal/basal border is depicted by a dotted white line. Bars, 20 µm. **(D)** Bar chart shows log_2_(fold change RNA abundance) for epigenetic proteins in EP-treated basal and luminal cells, determined by microarray (biological replicates, *n* = 4); asterisk denotes significantly altered genes (*q* < 0.05). **(E)** Bar charts show maximum normalized protein abundance of epigenetic proteins in ER^−^PR^−^ luminal progenitor and ER^+^PR^+^ luminal cells, taken from E- and EP-treated mice as determined by UPLC-MS (biological replicates, *n* = 2). *, P ≤ 0.05; **, P ≤ 0.01; ***, P ≤ 0.001; ****, P < 0.0001.

First, we performed an in-depth assessment of short-listed epigenetic proteins in the adult mammary gland via single-cell intracellular flow cytometry and in situ analyses. Our gating strategy with lineage marker controls is shown in Fig. S3 A. Consistent with our proteomics findings, intracellular flow cytometry revealed a luminal-basal disparity in some, but not all, epigenetic master-regulators. Specifically, EZH2, HDAC1, and G9a/EHMT2, but not HDAC2 and CREBBP, were significantly elevated in EP luminal compared with EP basal cells (Figs. 5 A and S3 B). Higher expression of epigenetic modifiers in luminal cells was hormone dependent, and only HDAC1 showed a significant luminal-basal difference in E-treated mice (Fig. 5 A). Several proteins (EZH2, HDAC1, HDAC2, CREBBP, and G9a/EHMT2) were increased by progesterone in both compartments (Figs. 5 A and S3 B). Among these, only EZH2 was previously shown to increase in the mammary gland with sex hormones (Pal et al., 2013). Therefore, progesterone broadly up-regulates components of the epigenetic machinery across the two mammary lineages.

Immunofluorescence (IF) spatially confirmed the aforementioned differences in protein expression, exposed the heterogeneous nature of many epigenetic proteins, and demonstrated that SETD7 but not GLP/EHMT1 is elevated in luminal cells (Fig. 5, B and C; and Fig. S3, B and C). It also revealed differential expression of DNMT enzymes across lineages; DNMT1 and DNMT3b are higher in luminal cells and DNMT3a in basal (Fig. 5 C). Microarray data showed similar expression patterns for most genes at the RNA level, with *Dnmt1* and *Dnmt3a* displaying significant differences (Fig. 5 D). Heterogeneous expression of epigenetic proteins in the luminal compartment corroborates mammary proteomics that shows enrichment of EZH2, HDAC2, CREBBP, DNMT1, and DNMT3a in ER^−^PR^−^ progenitors over ER^+^PR^+^ cells after EP treatment (Fig. 5 E). DNMT1 largely acts on hemimethylated CpGs during S-phase of the cell cycle, and this DNA methyltransferase displayed high overlap with proliferation marker Ki67 (Fig. 5 C). We also ascertained the status of mutually exclusive H3K27me3 (repressive) and H3K27ac (activating) chromatin marks, which are regulated by our target proteins. Both marks tended to be higher in the luminal compartment, with H3K27me3 exhibiting heterogeneous expression similar to EZH2 and being up-regulated by progesterone (Fig. 5, A and B; and Fig. S3 B). This systematic validation confirmed that specific epigenetic marks and regulators are increased by progesterone exposure.

### Epigenetic drugs effectively target basal and luminal mouse progenitor cells

**Figure 6.**
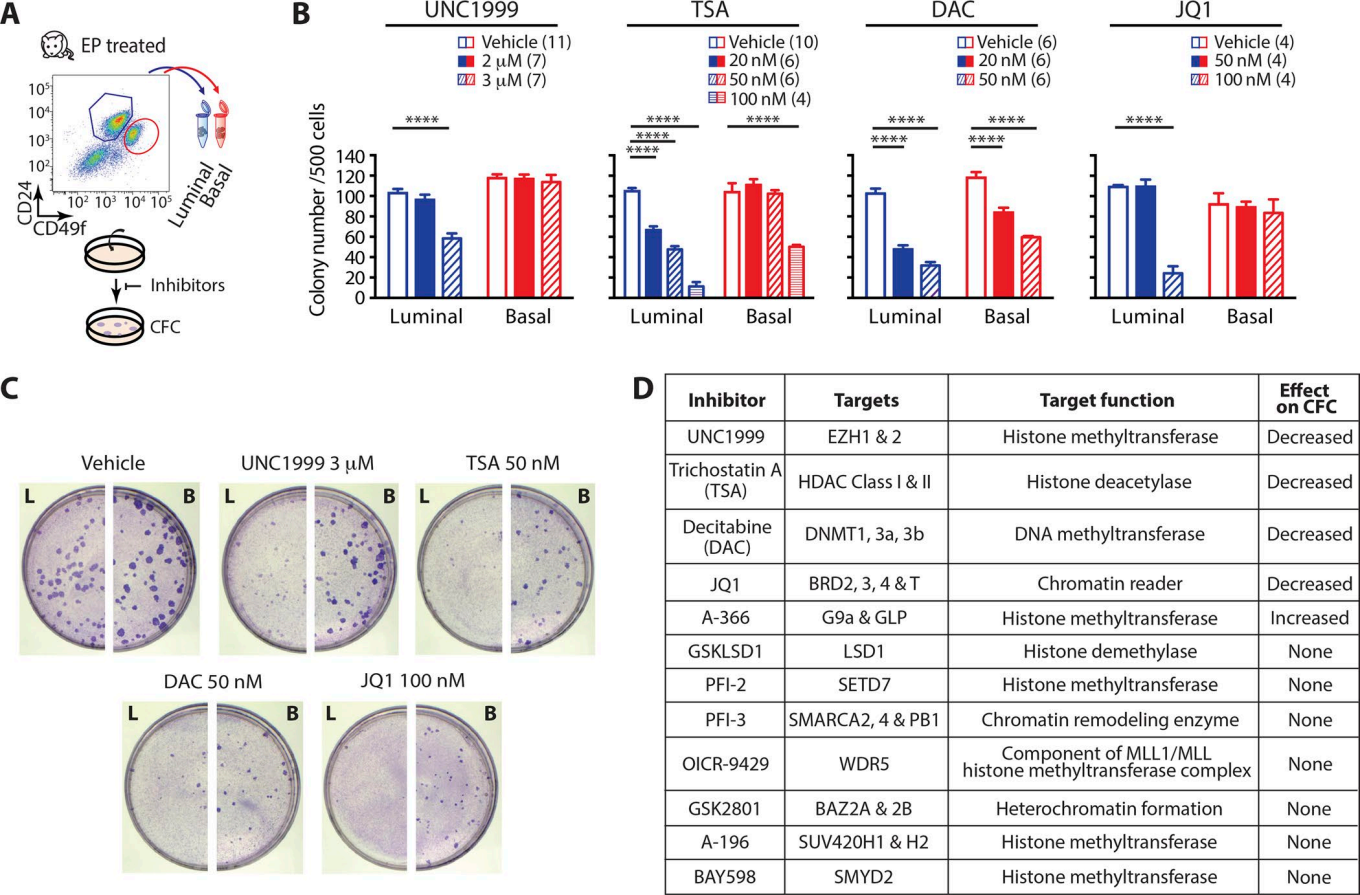
**Epigenetic targeting exposes mouse mammary progenitor cell vulnerabilities. (A)** Workflow schematic for epigenetic drug testing. **(B)** Bar charts show number of basal and luminal colonies formed with vehicle control or the indicated concentrations of epigenomic inhibitors. Number of biological replicates per drug treatment is shown in brackets. Error bars represent SEM. Statistical significance was calculated using two-way ANOVA and Dunnett’s multiple comparisons test performed with a 0.05 significance level and 95% confidence interval. Statistically significant differences are indicated by asterisks, which denote size of significance levels. ****, P < 0.0001. **(C)** Photographs of representative luminal (L) and basal (B) colony assay plates. **(D)** Summary of compounds tested and their targets and effects against mammary progenitor function.

We next asked whether epigenetic proteins are required for mammary progenitor function using Food and Drug Administration (FDA)–approved drugs or highly potent and selective chemical probes. We matched 12 drugs to rationalized epigenetic targets and examined their effects on FACS-purified basal and luminal cells in 2D clonogenic assays (Fig. 6 and Fig. S4, A and B). Each drug was individually titrated and used at low, physiologically relevant concentrations according to the literature or Structural Genomics Consortium (SGC) guidelines. Of these 12 compounds, seven had no effect, one increased basal CFC, and four robustly interfered with basal and/or luminal CFC (summarized in Fig. 6 D). Drugs and targets effective against mammary progenitors are: UNC1999 → EZH1 and 2; trichostatin A (TSA) → HDAC class I and II; DAC → DNMT1, 3a, 3b; and JQ1 → BRD2, 3, 4, and T (Fig. 6, B and C). These top four drugs are all FDA approved, are in phase I/II clinical trials, or have counterparts at similar stages of drug development. UNC1999, TSA, and JQ1 preferentially abrogated luminal over basal CFC (Fig. 6, B and C), with both JQ1 and TSA still reducing the size and number of basal colonies at nanomolar concentrations (Figs. 6 C and S4 A).

### Epigenetic drugs prevent progenitor cell expansion and mammopoiesis via cytostatic effect

**Figure 7.**
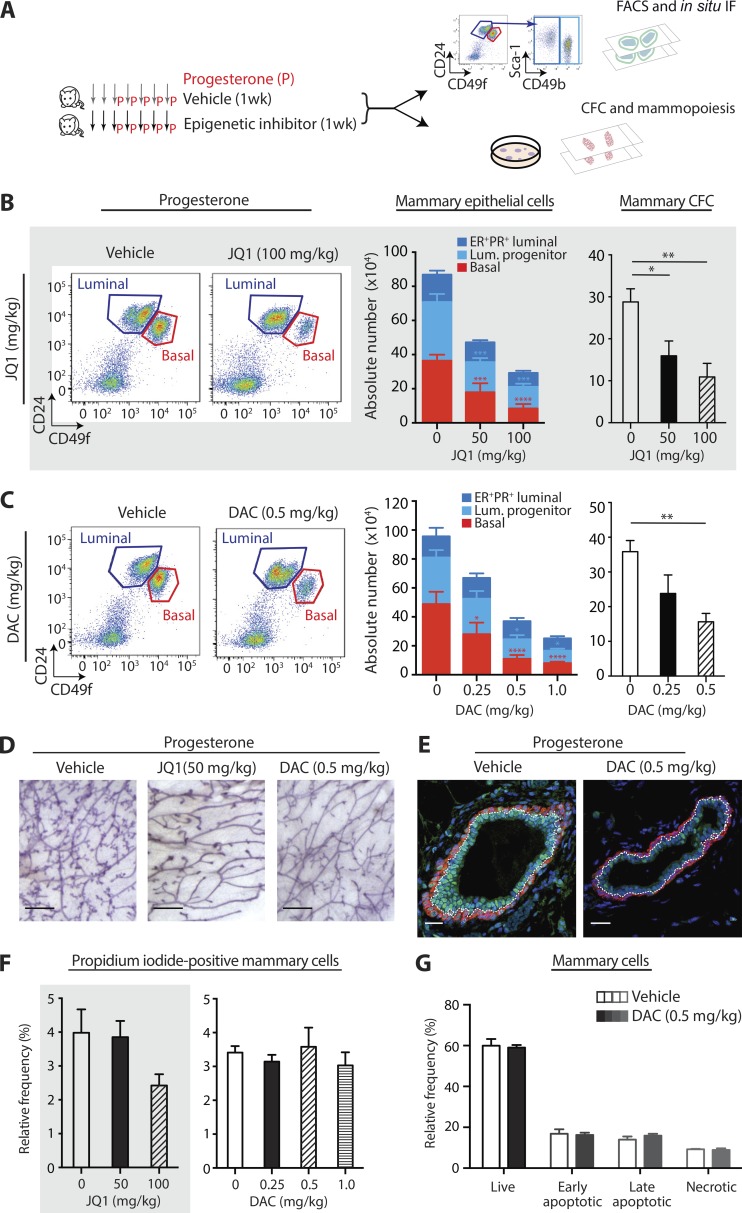
**DAC and JQ1 prevent adult progenitor cell expansion and mammopoiesis in vivo. (A)** Workflow schematic for in vivo drug testing. **(B and C)** Flow cytometry analysis of luminal (CD24^+^CD49f^lo^) and basal (CD24^−^CD49f^hi^) mammary subsets. Primary mammary cells were purified from the two inguinal glands of mice treated for 1 wk with vehicle, JQ1 (gray background), or DAC (clear background), + progesterone. Left: Bar charts show absolute number of ER^−^PR^−^ basal, ER^−^PR^−^ luminal progenitor, and ER^+^PR^+^ luminal cells, which were further purified using the CD49b and SCA-1 cell-surface markers. Right: Bar charts show absolute number of CFC. Error bars for all bar charts represent SEM. Statistical significance was calculated using one-way (absolute CFC) or two-way (absolute ER^−^PR^−^ basal, ER^−^PR^−^ luminal progenitor, and ER^+^PR^+^ luminal) ANOVA followed by Dunnett’s multiple comparisons test. All multiple comparisons testing was performed with a 0.05 significance level and 95% confidence interval; statistically significant differences are indicated by asterisks, which denote size of significance levels. In B, biological replicates: *n* ≥ 7 left stacked bar charts, *n* ≥ 4 right CFC bar chart; in C, biological replicates: *n* ≥ 4. **(D)** Representative whole mounts from mice treated with vehicle or indicated epigenetic drug. Bars, 1 mm. **(E)** IF staining of mammary ductal structures: DAPI (blue), basal lineage marker KRT14 (red), and DNMT1 (green). Luminal/basal border is depicted by a white dotted line. Bars, 20 µm. **(F)** Bar charts show relative frequency of primary mammary dead cells purified from the two inguinal glands of mice treated for 1 wk with JQ1 (gray background) or DAC, + progesterone. Dead cells were determined via propidium iodide staining; biological replicates: *n* ≥7 (JQ1) or *n* ≥4 (DAC). Statistical significance was tested for using one-way ANOVA followed by Dunnett’s multiple comparisons test; no comparisons were found to be statistically significant. **(G)** Bar charts show relative frequency of early- and late-apoptotic mammary cells after treatment with 0.5 mg/kg DAC, three weekly doses for 4 wk, determined via annexin V and propidium iodide staining. Biological replicates, *n* = 5. Statistical significance was tested using two-way ANOVA followed by Sidak’s multiple comparison test; no comparisons were found to be statistically significant. *, P ≤ 0.05; **, P ≤ 0.01.

Genetic studies have shown that conditional loss of *Dnmt1* or *Ezh2* compromises pubertal mammary gland development, resulting in truncated structures that possess fewer stem and progenitor cells (Michalak et al., 2013; Pal et al., 2013; Pathania et al., 2015). The importance of these and other epigenetic regulators in controlling adult stem cell pools has yet to be determined. We tested top epigenetic drugs in vivo to measure effects against stem- and progenitor-driven mammopoiesis in adult mice. We adapted our model to shorten the duration of sex hormone administration while preserving mammary cell expansion (Fig. 7 A). Each drug was given to mice for 1 wk along with progesterone, followed by mammary whole mounts, flow cytometry, and progenitor cell enumeration. Drugs tested were DAC, JQ1, and suberoylanilide hydroxamic acid (SAHA), the latter being an FDA-approved substitute for TSA (Fig. 7, B–D; and Fig. S4, C–E). Both DAC and JQ1 imparted dose-dependent inhibition of ER^−^PR^−^ basal and luminal progenitor expansion with a corresponding depletion in total mammary CFCs (Fig. 7, B and C; and Fig. S4 C). Mammopoiesis, as evident by side branching and alveoli formation, was also suppressed in drug-treated mice (Figs. 7 D and S4 D); these structures model the sites of breast cancer initiation in the human breast (Cardiff and Wellings, 1999). The HDAC inhibitor SAHA also prevented mammary epithelial expansion in response to progesterone, albeit at a higher concentration (Fig. S4 E). DAC exerted a cumulative effective on mammary progenitor inhibition, and administration of a low concentration over 2 or 4 wk significantly repressed mammary progenitor cell numbers (Fig. S4 F). Multicolor IF showed reduced DNMT1 and Ki67 positivity after 1 wk of DAC treatment (Figs. 7 E and S5 A).

1-wk treatment with DAC or JQ1 did not alter the frequency of mammary dead cells (Fig. 7 F). Further, absolute numbers of dead cells were lower, likely because drug-treated mice contained fewer total mammary epithelial and stromal cells (Fig. S5 B). DAC also had no effect on early- or late-apoptotic cells after 4 wk (Figs. 7 G and S5 C). We thus conclude that DAC and JQ1 do not kill mammary cells, but rather prevent ER^−^PR^−^ basal and luminal progenitor populations from responding to biological mitogenic triggers.

### Epigenetic drugs affect mammary stem cell frequency and cell cycle

**Figure 8.**
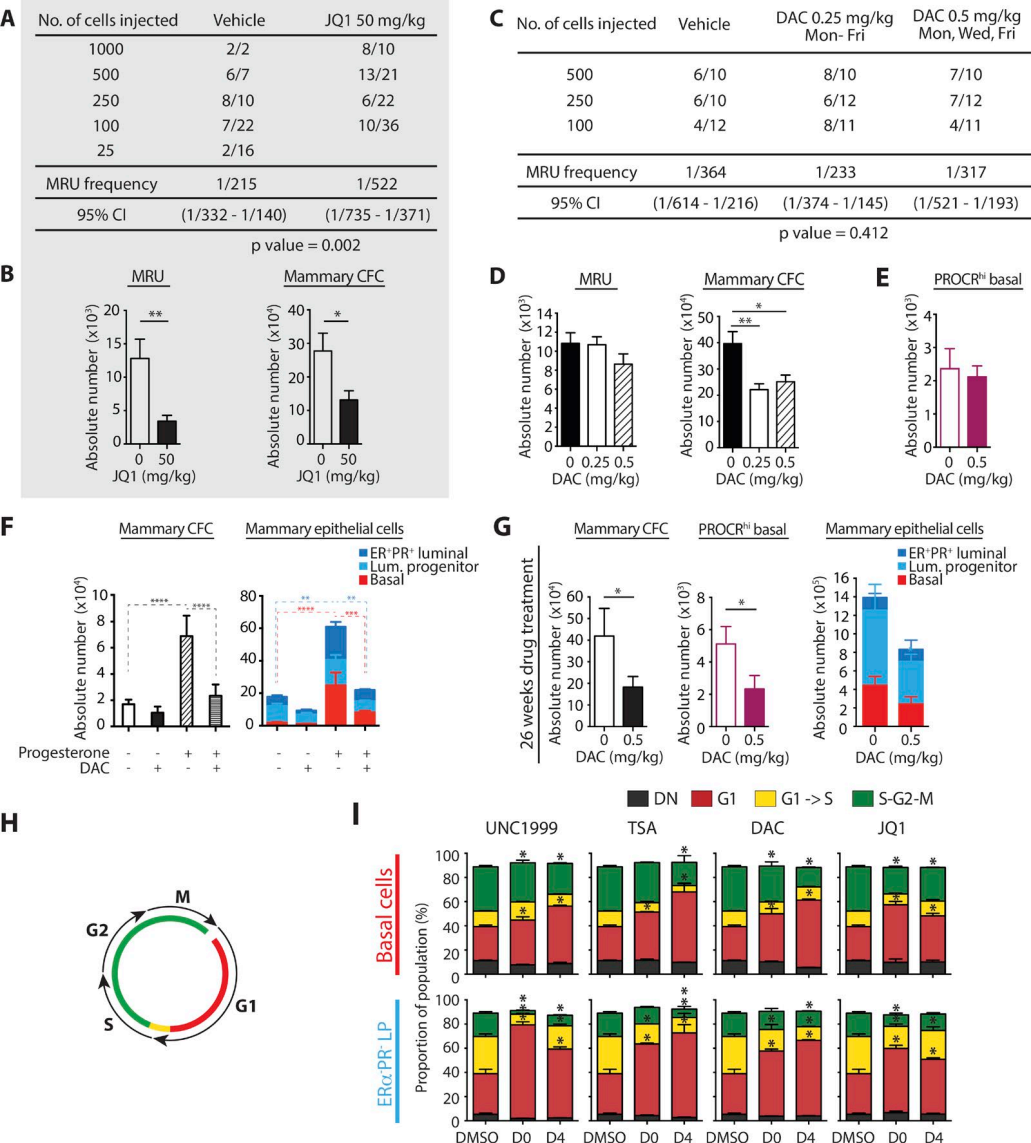
**Effects of epigenetic drugs on adult stem cell expansion and mammary cell cycle. (A and C)** Comparison of LDA take rates of total mammary cells purified from donor mice treated with vehicle or the indicated epigenetic inhibitors, + progesterone. Take rate is defined as a positive outgrowth. Cells were subsequently purified from the two inguinal glands for CFC assay. MRU frequencies for LDA experiments were calculated using ELDA software and a 95% confidence interval (Hu and Smyth, 2009). **(B and D)** Bar charts show absolute number of MRU and mammary CFC from the same total cell populations; all error bars represent SEM. Statistical significance was calculated using unpaired *t* test (*, P ≤ 0.05; **, P ≤ 0.01). **(A and B)** Donor mice were treated with progesterone + either vehicle or JQ1 (50 mg/kg, five weekly doses; gray background). Biological replicates: vehicle, *n* = 4; JQ1, *n* = 6. **(C and D)** Donor mice were treated with progesterone + either vehicle or DAC (0.25 mg/kg, five weekly doses; 0.5 mg/kg, three weekly doses; clear background). Biological replicates, *n* = 6. **(E)** Bar chart shows absolute number of PROCR^hi^ basal cells in mice treated with vehicle or DAC for 4 wk (0.5 mg/kg, three weekly doses); biological replicates, *n* = 5, error bars represent SEM. **(F)** Mice were treated with vehicle or 1 mg/kg DAC for 5 d + either sesame oil or progesterone. Biological replicates, *n* = 4; cells were purified from the two inguinal glands. Left: Bar chart shows absolute number of total mammary CFC. Right: Bar chart shows absolute number of ER^-^PR^-^ basal, ER^−^PR^−^ luminal progenitor, and ER^+^PR^+^ luminal cells. Error bars represent SEM. Statistical significance was calculated using one-way ANOVA (absolute CFC) or two-way ANOVA (absolute ER^−^PR^−^ basal, ER^−^PR^−^ luminal progenitor, and ER^+^PR^+^ luminal) followed by Tukey’s multiple comparisons test. **(G)** Mice were treated with vehicle or DAC for 26 wk (0.5 mg/kg, three weekly doses); biological replicates, *n* = 3. Bar charts shows absolute number of total mammary CFC (left), PROCR^hi^ basal cells (middle), and ER-PR- basal, ER-PR- luminal progenitor, and ER^+^PR^+^ luminal cells (right). Cells were purified from the two inguinal mammary glands; all error bars represent SEM. Statistical significance was calculated using unpaired *t* test (absolute CFC or PROCR^hi^ basal cells) or two-way ANOVA followed by Sidak’s multiple comparisons test. **(H)** Schematic of the Fucci2 reporter mouse transgenic system. **(I)** Basal or ER^−^PR^−^ luminal progenitor cells were FACS-sorted, plated in 2D clonogenic assays, and treated with vehicle or epigenetic drugs: 3 µM UNC1999, 50 nM TSA, 50 nM DAC, or 75 nM JQ1. Drugs were added on day 0 or 4 of a colony-forming assay, with bar charts showing the proportion of cells in different phases of the cell cycle at day 7, determined by flow cytometry. Biological replicates, *n* = 3; all error bars represent SEM. Statistical significance was calculated using two-way ANOVA followed by Dunnett’s multiple comparisons test. For all panels, all multiple comparisons testing was performed with a 0.05 significance level and 95% confidence interval. Statistically significant differences are indicated by asterisks, which denote P < 0.05. *, P ≤ 0.05; **, P ≤ 0.01.

Limiting dilution assays (LDAs) are used to measure stem cell frequency in multiple tissues, including the mammary gland (Hu and Smyth, 2009). We performed LDAs to enumerate mammary repopulating units (MRUs) in mice treated with top epigenetics drugs or vehicle controls for 4 wk, plus progesterone (Fig. 8, A–D; workflow schematic Fig. S5 D). JQ1 treatment (50 mg/kg, five weekly doses) significantly reduced the frequency and absolute number of MRUs, demonstrating its depletion of the adult mammary stem cell pool (Fig. 8, A and B). On the other hand, DAC treatment (0.25 mg/kg DAC, five weekly doses; 0.5 mg/kg DAC, three weekly doses) had no significant effect on MRU frequency or absolute number (Fig. 8, C and D). PROCR^hi^ basal cells also remained unaffected in DAC-treated mice (Fig. 8 E); these are reportedly enriched for bipotent mammary stem cells (Wang et al., 2015). Thus, both JQ1 and DAC significantly inhibit mammary cell clonogenicity, whereas JQ1 also depletes bipotent, mammary stem cells.

The hypomethylating agent DAC is FDA approved for myelodysplastic syndrome and has been subject to hundreds of phase I–IV clinical trials. We rationalized that DAC treatment may impose a cytostatic effect on the mammary gland, thereby blocking sex hormone–triggered epithelial cell expansion. Supporting this, DAC treatment depleted mammary epithelial/progenitor cells only in the presence of exogenous progesterone (Fig. 8 F). We also asked whether prolonged DAC administration could limit natural stem and progenitor expansions occurring in response to hormonal fluctuations of the reproductive cycle. This dynamic cellular turnover in the adult mammary gland is linked to breast cancer risk. Mice were treated with DAC for 26 wk (0.5 mg/kg, three weekly doses). Fig. 8 G shows that prolonged DAC treatment reduced the absolute number of ER^−^PR^−^ luminal progenitor cells, mammary CFC, and PROCR^hi^ basal cells.

We next determined how epigenetic drugs exert a cytostatic effect on mammary progenitor cells. We took advantage of the Fucci2 reporter mouse in which cells express mCherry-hCdt1 during G1 and mVenus-hGem during S/G2/M (Abe et al., 2013). This transgenic system allows precise quantification of cell cycle phases (modeled in Fig. 8 H), which we applied to clonogenic assays. Fig. 8 I shows that all four of our top epigenetic drugs, identified via multimodal mammary cell profiling, significantly increased the proportion of cells in G1 and decreased cells in G2, S, and M. Consistent with our previous observations (Fig. 6, B and C), the effect of UNC1999 and TSA on cell cycle progression was greater in luminal versus basal colonies (Fig. 8 I). Overall, epigenetic drugs prevent mammopoiesis and limit stem and progenitor expansion via stalling of mammary cells in G1.

### Epigenetic targeting of normal and high-risk human breast progenitor cells

**Figure 9.**
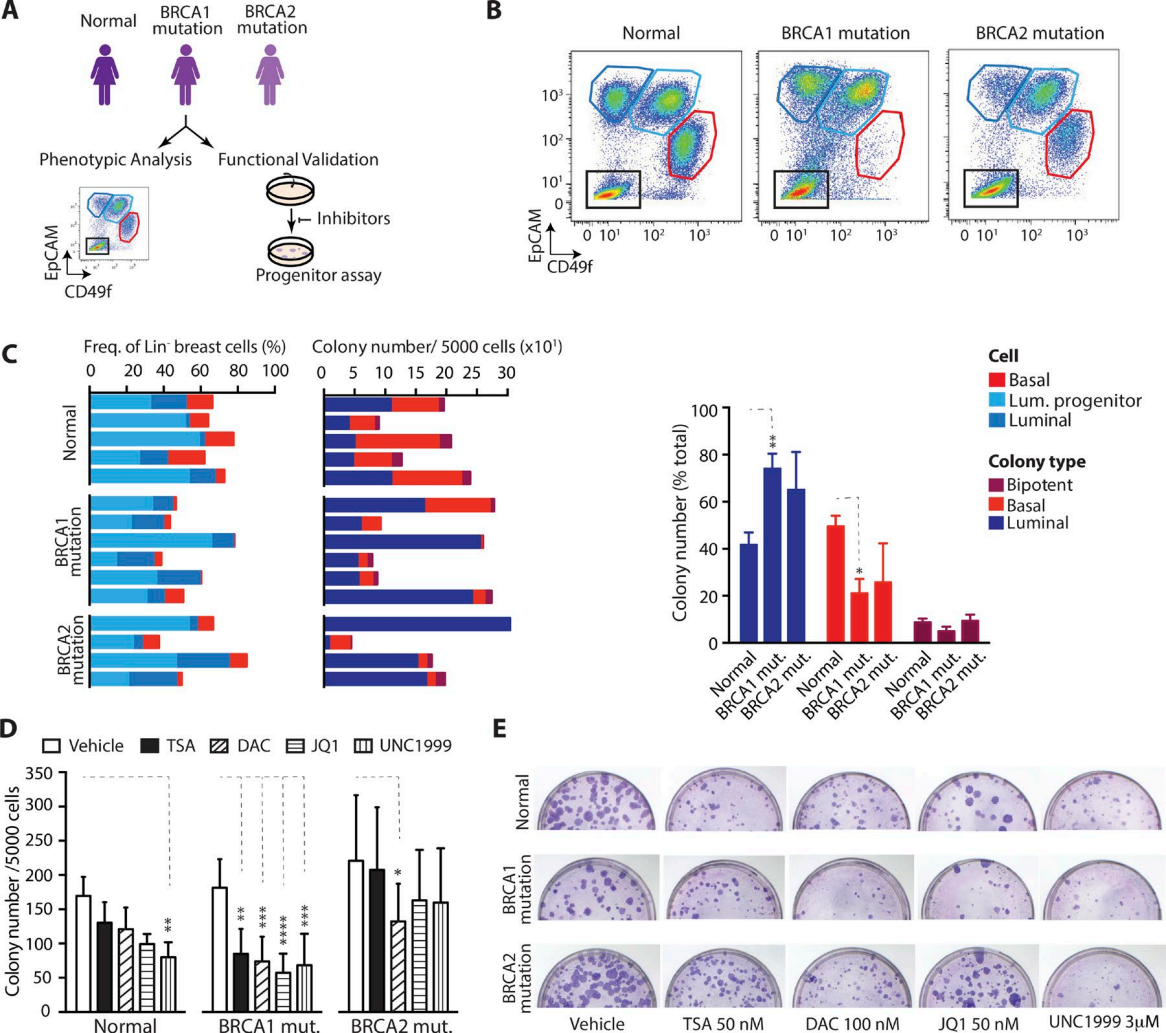
**Epigenetic targeting of normal and high-risk human breast progenitor cells. (A)** Workflow schematic. **(B)** Example flow cytometry plots of dissociated breast cells from normal and high-risk BRCA1 or BRCA2 mutation carrying patients. Plots show basal (EpCAM^-/low^CD49f^+^), luminal progenitor (EpCAM^+^CD49f^+^), and luminal (EpCAM^+^CD49f^−^) cells. **(C)** Left: Bar chart shows relative proportions of human basal (red), luminal progenitor (light blue), and luminal (darker blue) cells in samples from individual women, assayed by flow cytometry. Middle: Bar chart shows numbers and types of unsorted, total colonies formed from the same patient samples. Right: Bar chart shows percentage of colonies that are luminal, basal, or bipotent in different patient groups (biological replicates, *n* = 4–6). For bar chart on the right, error bars represent SEM, and statistical significance was calculated using two-way ANOVA and Tukey’s multiple comparisons test. **(D)** Bar chart shows number of colonies formed from normal and BRCA1 and BRCA2 mutation carrying patient specimens, treated with vehicle or the indicated concentrations of epigenetic inhibitors. Biological replicates, *n* = 4–6; error bars represent SEM. Statistical significance was calculated using two-way ANOVA and Dunnett’s multiple comparisons test. All multiple comparisons tests were performed with a 0.05 significance level and 95% confidence interval. Statistically significant differences are indicated by asterisks, which denote size of significance levels. **(E)** Photographs of representative colony assay plates. *, P ≤ 0.05; **, P ≤ 0.01; ***, P ≤ 0.001; ****, P < 0.0001.

An important step in ultimate clinical translation is to test whether drugs prove effective against human breast cells. We examined our shortlisted candidates using clinical specimens derived from normal and high-risk women. We obtained patient material from women undergoing reduction mammoplasty (BRCA wild-type) or prophylactic mastectomy (high-risk, BRCA1 or BRCA2 mutant). First, we measured the relative proportions of breast cell subpopulations—basal, luminal, luminal progenitor, and stromal—in tissues from each subject. (Fig. 9 A). The corresponding FACS profiles and progenitor enumeration reflected heterogeneity between women and the expected preponderance of luminal progenitors in BRCA1 mutation carriers (Fig. 9, B and C; Lim et al., 2009). Second, CFC for each patient specimen was enumerated with vehicle or epigenetic drugs. Colonies from normal women were significantly reduced by only one of the four inhibitors shortlisted from our mouse studies: UNC1999 (Fig. 9, D and E). In contrast, BRCA1 mutant cells were highly sensitive to epigenetic inhibition, with significant reduction of CFC by all four drugs, whereas BRCA2 mutant cells were less affected and susceptible only to the DNMT inhibitor DAC. It is noteworthy that primary cells from BRCA1 mutation carriers show sensitivity to several epigenetic drugs, and that DAC proved effective against specimens from both BRCA1/2 high-risk groups. These data demonstrate that progenitor cell dependence on specific epigenetic proteins is conserved between mice and humans and highlight the potential of epigenetic therapy to target pertinent cell compartments in the human breast.

### DAC delays mammary tumor formation in mice with conditional loss of *Trp53*

**Figure 10.**
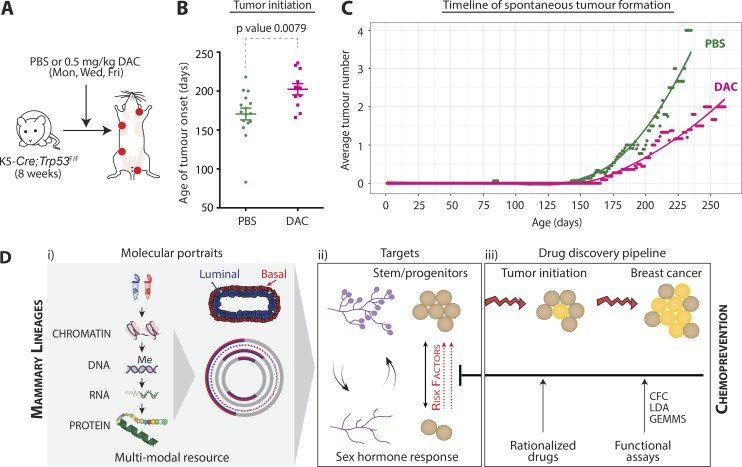
**DAC delays mammary tumor formation. (A)** Workflow schematic; red dots designate breast tumors at end point. **(B)** Graph shows time to first, spontaneous breast tumor formation in K5-*Cre*;*Trp53*^F/F^ mice treated with vehicle or 0.5 mg/kg DAC three times weekly, from 8 wk of age onward. Error bars represent SEM. Statistical significance was calculated using unpaired, two-tailed *t* test. **(C)** Graph shows mean number of palpable tumors in K5-*Cre*;*Trp53*^F/F^ mice treated with vehicle or DAC, plotted against age in days; shading shows local regression (Loess)-fitted smooth curve. Number of biological replicates for PBS- and DAC-treated mice, *n* = 16 and 11, respectively. **(D)** Model depicting how mammary molecular portraits can be used to garner new insight into the basal and luminal epithelial lineages, identify adult stem and progenitor vulnerabilities, and discover drug targets for breast cancer chemoprevention.

Next, we tested whether DAC could prevent breast cancer formation. We selected a *Trp53-*driven model because mutations in *TP53* occur frequently in breast cancer, are more common in BRCA1 or BRCA2 mutation carriers, and associate with aggressive tumors and adverse prognosis (Koboldt et al., 2012; Stunnenberg et al., 2016). In mice, loss of *Trp53* drives mammary tumorigenesis and results in cancers comprising a spectrum of molecular subtypes (Herschkowitz et al., 2012). Notably, deletion of *Trp53* in the mouse mammary epithelium also causes expansion of both luminal and basal stem and progenitor cells (Jackson et al., 2015). We used mice that have conditional deletion of *Trp53* in both mammary epithelial layers, via cytokeratin 5 (K5) promoter-driven Cre (K5*Cre*;*Trp53^F/F^*). Treatment of 8-wk-old, adult K5*Cre*;*Trp53^F/F^* mice with 0.5 mg/kg DAC, three times weekly, led to a marked delay in tumor initiation (P = 0.0079; Fig. 10, A–C). We observed multiple spontaneous mammary tumors in these mice, with a median onset of 172 d. The median number of tumors observed in K5*Cre*;*Trp53^F/F^* mice at end point was four. Further, age-matched, DAC-treated mice consistently had fewer palpable tumors throughout their lifespan. The only metastasis-bearing mouse was observed in the vehicle-treated group, in which metastases were found in the bone, lungs, and liver.

## Discussion

To generate fundamental information on the mammary epithelial lineages, we built a multimodal resource capable of querying genes at four separate levels of regulation. Another key objective was to model low- versus high-progesterone states and identify protein changes associated with stem and progenitor expansion and increased breast cancer risk. An important unknown is how the epigenome, transcriptome, and proteome landscapes work together to govern mammary cells. Two-way comparisons confirm a positive association between open chromatin, RNA, and/or protein abundance. Yet higher-order classification of genes based on their chromatin–DNA–RNA–protein relationship states reveal that most genes fall outside conventional patterns expected from the central dogma of molecular biology. For instance, we identified copious cell type–specific differences in open chromatin that did not amount to changes in RNA or protein. Further, DNA hypomethylation did not associate with higher gene expression, and a weak positive correlation between RNA and protein changes indicated substantial translational and posttranslational control. Of the 35% of genes exhibiting changes in RNA or protein abundance across basal and luminal cells, only 3% displayed the pattern of more open chromatin as well as increased RNA and protein abundance. Collectively, these findings highlight the intricate nature of cellular control over breast cell lineages and the value of a resource that enables researchers to query genes at successive levels of regulation.

Sex hormone exposure elevates breast cancer risk, with progesterone driving cyclical mammopoiesis events coupled to mammary stem and progenitor cell expansion (Joshi et al., 2010, 2015a). Proteomes for primary mammary subpopulations can now be mined to interrogate mitogenic effects of progesterone on ER^−^PR^−^ basal and luminal progenitor subsets, direct effects of progesterone on ER^+^PR^+^ luminal cells, and discover proteins responsible for functional changes in stem and progenitor activity. Visual composites of progesterone responses illustrate that more pathways are up-regulated in luminal progenitors, whereas a dramatic number of pathways are down-regulated in the basal compartment. Consistent with a mitogenic response, many pathways specific to ER^−^PR^−^ cells project nuclear changes associated with cell replication, DNA integrity, and epigenetic processes. Using discovery proteomics, we expose stem and progenitor cell vulnerabilities to highly specific and potent epigenetic drugs. Finally, we demonstrate feasibility of in-depth proteomics on ∼100,000 FACS-purified, primary cells which can be applied to rare cell fractions from other tissues.

Currently, there are no standard-of-care preventive interventions for women at high risk of breast cancer. Although it is becoming increasingly clear that stem and progenitor cells underlie cancer development (Visvader, 2011), strategies to target these cells for chemoprevention are lacking. We uncover lineage distinctions in epigenetic master regulators and demonstrate these proteins to be both hormonally regulated and required for progenitor function. Our results show that specific epigenetic drugs limit expansion of adult breast stem and progenitor cells in vivo, preventing mammopoiesis and depleting absolute numbers of these precursor cells. Additionally, treatment of mice with DAC and JQ1 did not cause detectable toxicity, but rather affected the cell cycle to exert a cytostatic effect on mammary cells, preventing them from responding to mitogenic stimuli.

Through mammary molecular portraits described herein, we build new capacity to identify promising avenues for chemoprevention (modeled in Fig. 10 D). We show that targeting epigenetic proteins inhibits clonogenicity of primary breast cells from women who carry BRCA1 or BRCA2 mutations. In particular, DAC is an FDA-approved compound used to treat myelodysplastic syndrome, making window-of-opportunity trials a realistic possibility. Prolonged treatment of mice with DAC over 26 wk countered cyclical expansions of stem and progenitor cells, which occur during natural reproductive cycles and are linked to breast cancer risk. DAC also delayed mammary tumor latency in conditional *Trp53* knockout mice. BET bromodomain inhibitors such as JQ1 are active against triple-negative breast cancer cell lines and xenografts (Shu et al., 2016); these aggressive cancers have poor prognosis and no targeted therapies and account for a high proportion of cancers arising in BRCA1 mutation carriers. Our finding that JQ1 is effective against adult mammary stem and progenitor cells opens the possibility for their use in targeting cells of origin in this disease.

## Materials and methods

### Mice

#### Husbandry

All experiments were performed according to guidelines from the Canadian Council for Animal Care and under protocols approved by the Animal Care Committee of the Princess Margaret Cancer Centre (Toronto, ON, Canada). Transgenic female K5-*Cre*;*Trp53*^F/F^ mice were bred in an FVB/C57BL6 background, and Fucci2 reporter mice were bred in a C57BL6 background. Virgin female FVB wild-type mice were purchased from either the Jackson Laboratory or Charles River. All experiments in wild-type mice were performed with FVB mice aged 8–12 wk. These mice had a mean weight of 22 g and were healthy and immune competent. Mice were housed in a modified barrier, specific pathogen–free facility in sealed negative ventilation cages (Allentown) with groups of two to five mice per cage, at 22°C–24°C and a 12-h light/12-h dark cycle with ad libitum food and water.

#### Primary cell sample preparation

Where indicated, mice were bilaterally ovariectomized and allowed to recover for 7 d. Mice were then subcutaneously implanted with slow-release hormone pellets, 0.14 mg 17β-estradiol (E) or 0.14 mg 17β-estradiol + 14 mg progesterone (EP; Innovative Research of America), and left for an additional 14 d.

#### In vivo dosing for mammopoiesis assays

For in vivo drug treatments, non-ovariectomized mice were randomly assigned to experimental groups. Mice then underwent daily subcutaneous injections with the indicated concentrations of DAC, SAHA, and JQ1 or the relevant vehicle controls for 1–4 wk as indicated in figure legends and workflow schematics. For dosing regimens lasting 2 or 4 wk, drugs were administered for five consecutive days followed by a 2-d break from injections. For the last 4–5 d of all drug treatments, mice were coinjected with 100 µl sesame oil containing 1 mg progesterone.

#### LDAs

Calculation of MRU frequencies by LDAs was performed as described previously (Joshi et al., 2010). Donor mice were treated with the indicated epigenetic inhibitor or vehicle control plus progesterone, as described above. Single-cell suspensions of total mammary cells from donor mice were resuspended in 10 µl Matrigel and DMEM/F12 (1:1) plus 5% Trypan blue (Sigma). The indicated cell numbers were injected into cleared, contralateral fourth inguinal fat pads of 21- or 22-d-old recipient FVB female mice. After 6 wk, mice were subcutaneously injected with 1 mg progesterone, daily for 7 d, to test side-branching and alveologenesis capabilities of outgrowths. Mammary glands were then dissected, and whole-mount analysis was performed. Fat pads were scored as positive or negative depending on the presence or absence of ductal outgrowths.

### Patient samples

All human tissue was acquired with patient consent and institutional research ethics board approval. Reduction mammoplasty and prophylactic mastectomy specimens were transferred from the operating room on ice within 24 h of surgery. Tissue was dissociated into organoids and cryopreserved as described previously (Labarge et al., 2013). In brief, tissue was manually minced and incubated in DMEM/F12 1:1 medium with 15 mM Hepes plus 2% BSA, 1% penicillin-streptomycin, 5 µg/ml insulin, 300 U/ml collagenase (C9891; Sigma), and 100 U/ml hyaluronidase (H3506; Sigma) with gentle shaking at 37°C, overnight or for 16 h. Tissue organoids were harvested by washing with warm DMEM and spinning at 80 *g* for 30 s.

Tissue was from normal women with no known genetic history: *n* = 5; ages 27, 37, 38, 38, and 52 yr; high-risk women with germline BRCA1 mutations: *n* = 6; ages 26, 27, 31, 48, and 52 yr, and unknown; and high-risk women with germline BRCA2 mutations: *n* = 4; ages 39, 41, 43, and 47 yr.

### Primary cell colony-forming assay

#### Mouse CFC assay

All murine 2D colony-forming assays were performed using female FVB wild-type mice. In brief, total dissociated mammary cells or FACS-purified basal and luminal cells were seeded together with irradiated NIH 3T3 cells. Cells were allowed to adhere, and either vehicle control (0.1% DMSO) or the indicated concentrations of epigenomic inhibitor were added. Cells were cultured for 7 d at 5% oxygen to allow basal colony growth in either Epicult-B mouse medium (Stem Cell Technologies) supplemented with 5% FBS, 10 ng/ml EGF, 20 ng/ml basic FGF, 4 µg/ml heparin, and 5 µM ROCK inhibitor (Millipore) or DMEM/F12 (1:1) supplemented with 10% FBS, 5 µg/ml insulin (Thermo Fisher Scientific), 10 ng/ml EGF, 10 ng/ml cholera toxin, 1.8 × 10^4^ M adenine (Sigma), 0.5 µg/ml hydrocortisone, and 10 µM ROCK inhibitor (Millipore). Growth factors and hydrocortisone were obtained from Stem Cell Technologies.

#### Human CFC assay

Breast colony-forming assays were performed with patient samples as described previously (Eirew et al., 2010). Total dissociated breast cells were seeded with irradiated NIH 3T3 cells onto collagen-coated plates (04902; Stem Cell Technologies). Cells were cultured at 5% oxygen in Epicult-B human medium (Stem Cell Technologies) for 14 d. Cells were seeded for 24 h in medium containing 5% FBS. Medium was then changed to serum-free medium, and drugs or vehicle controls (0.1% DMSO) were added.

### Epigenetic drugs and chemical probes

#### Drug testing in vitro

Unless otherwise stated, epigenetic inhibitors or chemical probes were obtained from the SGC, an open-access organization with information related to their compounds available online. Links for compounds used in this study can be found in Table 1. For in vitro studies, UNC1999 (EZH1 and 2), SGCCBP30 (EP300 and CREBBP), JQ1 (BRD2, 3, 4 and T), A-366 (GLP/EHMT1 and G9a/EHMT2), GSKLSD1 (KDM1A/LSD1), PFI-2 (SETD7), PFI-3 (SMARCA2 and SMARCA4), OICR-414 (WDR5), TSA (HDAC class I and II; Sigma), and decitabine (DAC; DNMT1, 3a and 3b; Sigma) were dissolved in DMSO. Vehicle or drugs were added such that the final concentration of DMSO did not exceed 0.1% (vol/vol).

**Table 1.** SGC chemical probes: Links to selectivity, cell-based assay, and crystal structure information

Drug	Target	SGC link
UNC1999	EZH1 and 2	https://www.thesgc.org/chemical-probes/UNC1999
JQ1	BRD2, 3, 4 and T	https://www.thesgc.org/chemical-probes/JQ1
A-366	G9a and GLP	https://www.thesgc.org/chemical-probes/A-366
GSKLSD1	LSD1	https://www.thesgc.org/chemical-probes/GSK-LSD1
PFI-2	SETD7	https://www.thesgc.org/chemical-probes/PFI-2
PFI-3	SMARCA2, 4 and PB1	https://www.thesgc.org/chemical-probes/PFI-3
OICR-9429	WDR5	https://www.thesgc.org/chemical-probes/OICR-9429
GSK2801	BAZ2A and 2B	https://www.thesgc.org/chemical-probes/GSK2801
A-196	SUV420H1 and H2	https://www.thesgc.org/chemical-probes/A-196
BAY598	SMYD2	https://www.thesgc.org/chemical-probes/BAY-598

#### Drug testing in mice

DAC (Sigma) was dissolved in PBS; JQ1, in 5% (vol/vol) DMSO in 10% (wt/vol) hydroxypropyl-β-cyclodextrin (Sigma); and Vorinostat (SAHA; Cayman Chemicals), in DMSO.

### Mammary cell preparation and FACS

#### Mouse mammary single-cell suspensions

For murine cells, mammary glands were manually minced with scissors or scalpels for 2 min, then enzymatically dissociated using 750 U/ml collagenase and 250 U/ml hyaluronidase (Stem Cell Technologies) diluted in DMEM/F12 (1:1). Samples were vortexed after 1 and 1.5 h. Single-cell suspension preparation and cell sorting was performed as described previously (Joshi et al., 2010). In brief, red blood cell lysis was performed using ammonium chloride solution (Stem Cell Technologies). Cells were triturated in trypsin-EDTA (0.25%; Stem Cell Technologies) that had been prewarmed to 37°C, using a P1000 pipette for 2 min. Cells were then washed in HBSS without calcium or magnesium, plus 2% FBS, and centrifuged. Cells were similarly triturated in dispase 5 U/ml diluted in HBBS (Stem Cell Technologies) plus 50 µg/ml DNase I for 2 min, washed in HBBS + 2% FBS, and filtered using a 40-µM cell strainer.

#### Mouse mammary FACS staining

For FACS, staining antibodies were rat anti-mouse TER119 (clone TER-119; eBioscience); rat anti-mouse CD31 (PECAM-1, clone 390; eBioscience); rat anti-mouse CD45 (clone 30-F11; eBioscience); rat anti-mouse CD24 (clone M1/69; eBioscience); rat anti-mouse CD326 or EpCAM (clone G8.8; BioLegend); rat anti-human/mouse CD49f (Clone GoH3; BioLegend); Armenian hamster anti-CD49b (Clone HMα2; BioLegend); and rat anti–SCA-1 (clone D7; BioLegend). Mammary cell subpopulations were defined as basal, Ter119^−^CD31^−^CD45^−^CD24^lo-med^CD49f^hi^; luminal, Ter119^−^CD31^−^CD45^−^ CD24^hi^CD49f^lo^; luminal progenitor, Ter119^−^CD31^−^CD45^−^CD24^hi^CD49f^lo^CD49b^+^Sca-1^−^; and ERα^+^PR^+^ luminal cells, Ter119^−^CD31^−^CD45^−^ CD24^hi^CD49f^lo^CD49b^−^ Sca-1^−/+^ and Ter119^−^CD31^−^CD45^−^ CD24^hi^CD49f^lo^CD49b^+^Sca-1^+^. For flow cytometry analysis, dead and apoptotic cell frequencies were determined after doublet exclusion, using propidium iodide (10 µg/ml) and the annexin V apoptosis detection kit eFluor 450 (Invitrogen) according to the manufacturer’s instructions.

#### Human breast single-cell suspensions

Human breast tissue organoids were thawed and dissociated into single-cell suspensions as reported previously (Eirew et al., 2010). In brief, organoids were triturated in trypsin-EDTA (0.25%; Stem Cell Technologies) followed by dispase 5 U/ml and 50 µg/ml DNase I as described above for mouse samples, but for 5 min each. Cells were then washed in HBBS + 2% FBS and filtered using a 40-µM cell strainer.

#### Human breast FACS staining

Human breast cells were stained using the following antibodies: mouse anti-human CD45 (Clone HI30; BioLegend); mouse anti-human CD31 (Clone WM59; BioLegend); mouse anti-human EpCAM (Clone 9C4; BioLegend); and rat anti-human/mouse CD49f (Clone GoH3; BioLegend).

### Mammary cell intracellular flow cytometry

For intracellular flow cytometry analyses, single-cell suspensions of mouse mammary cells were stained with cell-surface markers described above. Cells were then washed and labeled with either LIVE/DEAD Fixable Far Red stain (Thermo Fisher Scientific) or Zombie UV Fixable Viability kit (BioLegend) according to manufacturer instructions. Cells were then fixed in 4% PFA dissolved in PBS, for 10 min at room temperature, washed, and permeabilized in 0.1% Triton X-100 for 5 min at room temperature. Cells were next incubated with the indicated Fc isotype control or intracellular primary antibody for 30 min at 4°C, washed twice in HBSS, and incubated for a further 30 min at 4°C with goat anti–rabbit IgG (H+L) secondary antibody conjugated to Alexa Fluor 488 (Thermo Fisher Scientific). All flow cytometry analysis was performed using a BD Biosciences Fortessa. Adjusted median fluorescent intensity (MFI) for each intracellular staining of each mammary population is defined as MFI(protein of interest) − MFI(Fc control).

Only antibodies with a stronger signal than their concentration-matched Fc isotype control were used for intracellular flow cytometry: rabbit anti-H3K27me3 (Clone C36B11; Cell Signaling); rabbit anti-EZH2 (Clone D2C9; Cell Signaling); rabbit anti-EHMT2 (Clone C6H3; Cell Signaling); rabbit anti-HDAC1 (ab53091; Abcam); rabbit anti-HDAC2 (Clone Y461; Abcam); rabbit anti-CREBBP (Clone D6C5; Cell Signaling); rabbit anti-cytokeratin 5 (K5; Clone EP1601Y; Abcam); and rabbit anti-K8 (LS-B12422; LifeSpan BioSciences). For controls, rabbit polyclonal IgG isotype control (Abcam) and rabbit mAb IgG isotype control (Cell Signaling) were used.

### Mammary tumor latency

K5-*Cre*;*Trp53*^F/F^ mice were injected subcutaneously with PBS or 0.5 mg/kg DAC three times weekly, from 8 wk of age onward. Mice were also weighed and checked for tumors via physical examination and palpation. A low frequency of mice treated with DAC (20%, 3/15) developed eye infections and were treated with enrofloxacin (Baytril, 25 mg/kg) and erythromycin eye ointment (Pendopharm); this side effect is consistent with known immunosuppressive properties of DAC and was not observed in any wild-type mice, indicating it is specific to the K5-*Cre*;*Trp53*^F/F^ strain. A low frequency of K5-*Cre*;*Trp53*^F/F^ mice, in both vehicle and drug-treated groups, also developed skin or oral invasive squamous cell carcinomas, indicating that *Trp53* expression is lost in other epithelial tissues.

### MS

Counts for mammary cell subpopulations isolated for UPLC-MS ranged from 66,000 to 240,000 cells. For initial UPLC-MS of basal versus luminal subsets, cells were FACS-purified from single EP-treated mice. For UPLC-MS of basal, ERα^−^PR^−^ luminal progenitor, and ERα^+^PR^+^ luminal cells (E vs. EP), equal numbers of cells were pooled from multiple mice. After FACS purification, cells were washed in ice-cold PBS and pelleted. Cell pellets were resuspended in 50% (vol/vol) 2,2,2-trifluoroethanol in PBS and disrupted into cellular lysates sequentially by repeated probe sonication, followed by six freeze-thaw cycles. Proteins in cellular lysate were denatured by incubation at 60°C for 2 h, and oxidized cysteines were reduced using 5 mm dithiothreitol for 30 min at 60°C and alkylated through reaction with 25 mM iodoacetamide for 30 min at room temperature in the dark. Each sample was diluted five times using 100 mM ammonium bicarbonate, pH 8.0. Proteins in lysates were digested into peptides by addition of 5 µg of MS-grade trypsin (Promega). The digestion was performed overnight at 37°C and subsequently desalted using OMIX C18 pipette tips (Agilent). Peptides were semidried through vacuum centrifugation and resuspended in water with 0.1% formic acid. Subsequently, all samples were analyzed using an Easy-LC1000 (Thermo Fisher Scientific) coupled to a QExactive tandem mass spectrometer (Thermo Fisher Scientific). Peptides were separated on a ES803 (Thermo Fisher Scientific) nano-flow column heated to 50°C using a 4-h reverse-phase gradient.

### Proteome bioinformatics

The obtained raw files were searched using MaxQuant (v.1.4.1.2) and a mouse UniProt sequences FASTA database (v.19-07-2012; number of sequences, 16,548). Carbamidomethylation of cysteines was defined as a static modification and oxidation of methionine as a variable modification. All searches were performed with 1% peptide spectral match and protein FDRs. Protein groups identified with at least two peptides were carried forward for additional analysis. When present for all samples, label-free quantification (LFQ) values produced from the MaxLFQ algorithm in MaxQuant were used as a measure of protein abundance (Cox et al., 2014). For proteins missing an LFQ value for one or more samples, LFQ intensities were replaced with median-adjusted intensity-based absolute quantification (iBAQ) values (Wojtowicz et al., 2016). Values were averaged for any technical replicates (indicated in Table S7 by “rep”). For proteins/subpopulations in which both LFQ and iBAQ values were missing, 0 values were replaced with 1. Normalized protein abundances were calculated by dividing each value for a protein by the maximum value observed in any sample.

Correlations between log2(RNA fold change) and log2(protein fold change) were calculated in the R statistical environment (v.3.1.0) with the cor function using the Spearman method. All Venn diagrams were made using VennDIS and depict proteins identified in distinct mammary cell compartments or up- and down-regulated by EP treatment (≥2-fold change, P < 0.05; Ignatchenko et al., 2015). Heatmaps were generated using Pearson correlation and unsupervised divisive hierarchical clustering and depict z-score–normalized protein expression across mammary subsets; for total proteome heatmaps, data were log_2_-converted. All z-scores were calculated in the R statistical environment (v.3.1.0) and represent (*x* − mean)/SD. Volcano plot x-axis shows log_2_(protein fold change), and y-axis, log_10_(adjusted p-values); p-values were calculated using unpaired *t* test.

Pathway analysis for proteomic datasets was performed using GSEA (Subramanian et al., 2005). For protein groups identified by UPLC-MS, only one protein per group was included. Pathways were excluded that contained fewer than 15 or more than 500 genes. For background genes, the total list of proteins identified in mammary proteomes was used. GSEA results were summarized using the Enrichment Map app (v.3.0) in Cytoscape (v.3.5.1). Each circle (node) represents a biological term, with node size being proportional to the number of associated genes. Nodes were organized into themes (larger, labeled circles) using the AutoAnnotate app, and theme names were manually edited (Shannon et al., 2003; Merico et al., 2010; Cerami et al., 2011; Kucera et al., 2016).

### Methylome analysis and bioinformatics

Libraries for RRBS were prepared as described previously (Gu et al., 2011), using 25,000 luminal and basal cells FACS-purified from individual EP-treated mice. RRBS data were analyzed with the package Bismark, whose output was further processed by methylKit to get the DMCs (Krueger and Andrews, 2011; Akalin et al., 2012). When comparing basal and luminal cells, DMCs were defined as having a delta-β of 0.15 and an adjusted p-value of <0.01. Heatmap depicts β-values and was generated based on 15% DMCs that had the highest variance across all four samples. Heatmaps were made using Euclidean distance and unsupervised average hierarchical clustering.

Transcription factor motif analysis was performed using the findMotifsGenome tool of HOMER v.4.7 (Heinz et al., 2010). Details regarding motifs and related transcription factors hypomethylated in luminal versus basal cells can be found in Tables 2 and 3. Enrichment scores for TFBS analyses were calculated as the log_2_ ratio of percentage target sequences, with the motif divided by percentage of background sequences with the motif. Genes proximal to hypomethylated TFBSs were annotated using the annotatePeaks tool of the HOMER package. Input for the function consisted of mouse genome (mm9) coordinates of the 300 bp around each hypomethylated DMC, which served as peaks, and a list of hypomethylated TFBS motifs to find. Annotated genes were then filtered to include only protein-coding genes, localized within 250 bp of ATAC-seq peaks (shared or lineage-restricted) and present in microarray data.

**Table 2.** Luminal cells: Identified HOMER motifs and corresponding transcription factor names

HOMER motif	Name	h-Me	ATAC-seq
FOXA1(Forkhead)/MCF7-FOXA1-ChIP-Seq	FOXA1	Yes	Yes
ETS1(ETS)/Jurkat-ETS1-ChIP-Seq	ETS1	Yes	No
Jun-AP1(bZIP)/K562-cJun-ChIP-Seq	JUN	Yes	No
AP-1(bZIP)/ThioMac-PU.1-ChIP-Seq(GSE21512)	FOS	Yes	No
Ets1-distal(ETS)/CD4+-PolII-ChIP-Seq	ETS1-distal	Yes	No
ERG(ETS)/VCaP-ERG-ChIP-Seq	ERG	Yes	No
Foxa2(Forkhead)/Liver-Foxa2-ChIP-Seq	FOXA2	Yes	Yes
NF1(CTF)/LNCAP-NF1-ChIP-Seq	NFIB	Yes	Yes
Foxo1(Forkhead)/RAW-Foxo1-ChIP-Seq	FOXO1	Yes	No
ELF5(ETS)/T47D-ELF5-ChIP-Seq(GSE30407)	ELF5	Yes	Yes
NF1:FOXA1/LNCAP-FOXA1-ChIP-Seq(GSE27824)	NF1:FOXA1	Yes	Yes
PU.1-IRF(ETS:IRF)/Bcell-PU.1-ChIP-Seq(GSE21512)	SPI1-IRF	Yes	No
PU.1(ETS)/ThioMac-PU.1-ChIP-Seq(GSE21512)	SPI1	Yes	Yes
Bach2(bZIP)/OCILy7-Bach2-ChIP-Seq(GSE44420)	BACH2	Yes	No
FOXP1(Forkhead)/H9-FOXP1-ChIP-Seq(GSE31006)	FOXP1	Yes	Yes
NF1-halfsite(CTF)/LNCaP-NF1-ChIP-Seq	NF1-halfsite	Yes	No
Fox:Ebox(Forkhead:HLH)/Panc1-Foxa2-ChIP-Seq(GSE47459)	FOX:EBOX	Yes	No
AP2gamma(AP2)/MCF7-TFAP2c-ChIP-Seq	TFAP2C	Yes	No
Bach1(bZIP)/K562-Bach1-ChIP-Seq(GSE31477)	BACH1	Yes	No
MyoD(HLH)/Myotube-MyoD-ChIP-Seq	MYOD1	Yes	No
MYB(HTH)/ERMYB-Myb-ChIPSeq(GSE22095)	MYB	Yes	No
Nrf2(bZIP)/Lymphoblast-Nrf2-ChIP-Seq(GSE37589)	NFE2L2	Yes	No
Pbx3(Homeobox)/GM12878-PBX3-ChIP-Seq	PBX3	Yes	No
AP-2alpha(AP2)/Hela-AP2alpha-ChIP-Seq	TFAP2A	Yes	No
TR4(NR/DR1)/Hela-TR4-ChIP-Seq	NR2C2	Yes	No
GATA-IR3(Zf)/iTreg-Gata3-ChIP-Seq(GSE20898)	GATA3	Yes	Yes

**Table 3.** Basal cells: Identified HOMER motifs and corresponding transcription factor names

HOMER motif	Name	h-Me	ATAC-seq
p63(p53)/Keratinocyte-p63-ChIP-Seq	TP63	Yes	Yes
p53(p53)/Saos-p53-ChIP-Seq	TP53	Yes	Yes
NF1-halfsite(CTF)/LNCaP-NF1-ChIP-Seq	NF1-halfsite	Yes	No
TEAD4(TEA)/Tropoblast-Tead4-ChIP-Seq(GSE37350)	TEAD4	Yes	No
Pax8(Paired/Homeobox)/Rat-Pax8-ChIP-Seq	PAX8	Yes	No
Tcf4(HMG)/Hct116-Tcf4-ChIP-Seq	TCF4	Yes	No
AR-halfsite(NR)/LNCaP-AR-ChIP-Seq(GSE27824)	AR-halfsite	Yes	No
X-box(HTH)/NPC-H3K4me1-ChIP-Seq	X-BOX(HTH)	Yes	No
TCFL2(HMG)/K562-TCF7L2-ChIP-Seq(GSE29196)	TCF7L2	Yes	No
NF1(CTF)/LNCAP-NF1-ChIP-Seq	NFIB	Yes	No
Ap4(HLH)/AML-Tfap4-ChIP-Seq(GSE45738)	TFAP4	Yes	No
Rfx5(HTH)/GM12878-Rfx5-ChIP-Seq(GSE31477)	RFX5	Yes	No
Egr2/Thymocytes-Egr2-ChIP-Seq(GSE34254)	EGR2	Yes	Yes
NFAT(RHD)/Jurkat-NFATC1-ChIP-Seq	NFATC1	Yes	No
EGR(Zf)/K562-EGR1-ChIP-Seq	EGR1	Yes	Yes
TEAD(TEA)/Fibroblast-PU.1-ChIP-Seq	TEAD	Yes	No
Atoh1(bHLH)/Cerebellum-Atoh1-ChIP-Seq	ATOH1	Yes	No
NFkB-p65(RHD)/GM12787-p65-ChIP-Seq	RELA	Yes	No
PPARE(NR/DR1)/3T3L1-Pparg-ChIP-Seq	PPARG	Yes	No
Tcf3(HMG)/mES-Tcf3-ChIP-Seq	TCF3	Yes	No
AP-1(bZIP)/ThioMac-PU.1-ChIP-Seq(GSE21512)	FOS	No	Yes
Jun-AP1(bZIP)/K562-cJun-ChIP-Seq	JUN	No	Yes

To calculate likelihood of methylation, DMCs hypomethylated in basal (or luminal) cells were overlapped with genic (promoters, exon, intron, and intergenic) or CGI-related (CGIs, shelfs, shores, and open-sea) features to get the “observed” number of overlaps. These DMCs were then randomly shuffled across the background set of CpGs (those present in all samples) 1,000 times and overlapped with features again to get the “expected” number of overlaps. These observed and permutation-based overlaps were used to compute p-values for enrichment/depletion using the R function pnorm, with the parameters mean and SD set to the mean and standard deviation, respectively, of the permutation-based values, and lower.tail set to “false” if the observed number of overlaps is higher than the mean of the expected overlaps; parameter log is set to “true” to return the natural log of the p-value, and then the actual p-value is calculated as the exponent of the returned value; R is unable to compute this if the returned value is less than −745, in which case the p-value is simply denoted as “e^(natural log of the p-value).” The fold change for likelihood of hypomethylation was calculated as observed overlaps divided by the mean of expected overlaps. If this value is less than 1, fold change is transformed to be its negative reciprocal. Positive fold change indicates enrichment of the feature, and negative fold change indicates depletion of the feature.

### ATAC-seq analysis and bioinformatics

Libraries were prepared using the NeBNext buffer and appropriate ATAC-seq libraries adaptors (Illumina; Buenrostro et al., 2013). The library was size-selected using DNA-on-a-ChIP technology. Reads were filtered for quality and trimming using FASTX Toolkit 0.0.13.1. Filtered reads were aligned to the mouse genome (mm10) using BWA (Li and Durbin, 2009). Duplicate reads were marked and removed using Picard (https://github.com/broadinstitute/picard). Peaks were called using MACS2.0 (https://github.com/taoliu/MACS/; Zhang et al., 2008). Transcription factor motif analysis was performed using HOMER v.4.7 on ATAC-seq peaks found only in basal or luminal cells (Heinz et al., 2010). Details regarding motifs and related transcription factors enriched in open chromatin found only in luminal or basal cells can be found in Tables 2 and 3. GREAT pathway analysis was performed on lineage-restricted ATAC-seq peaks, using total ATAC-seq peaks detected across both mammary lineages as background. Associated genomic regions were interrogated based on the two nearest genes with 50-kb maximum extension.

### Microarray analysis and bioinformatics

Microarray data were preprocessed as described previously (Shiah et al., 2015). In brief, raw intensities were background-corrected using normexp with offset 50. Loess and scale normalization was applied within and between arrays, respectively. Univariate linear modeling was conducted to identify genes with significant differential mRNA abundance levels between basal and luminal cells after EP treatment using the BioConductor package limma (v.3.26.9; Smith, 2005) in the R statistical environment (v.3.2.5). All model-based *t* tests were corrected using empirical Bayes moderation to reduce standard error (Smyth, 2004). FDR adjustments were applied to all p-values for multiple testing corrections (Storey and Tibshirani, 2003).

### Integrated analysis

For tabular comparisons, criteria for genes altered across basal and luminal cells were defined as follows: protein up-regulation (≥2-fold change, P < 0.05), mRNA up-regulation (*q* < 0.05), and lineage-restricted ATAC-seq peaks (detected in only one of the two cell types). Hyper- and hypomethylated DMCs were defined as described above (delta-β of 0.15 and adjusted P < 0.01). ATAC-seq peaks and DMCs were annotated to a gene if their coordinates were within a region of 2,500 bp upstream of the gene start and end position. For DMCs, promoter methylation refers to coordinates upstream of TSS, and genic methylation refers to coordinates inside the gene body.

For integrated heatmap and barplot, genes with overlapping data for all types of molecular analyses (*n* = 3,424) were assigned a relationship state. Each relationship state contains ternary information (up in basal, up in luminal or neutral) for open chromatin, DNA methylation, RNA abundance, and protein abundance. For open chromatin, genes were classified as increased if the number of ATAC-seq peaks was greater in one cell type versus another. Peak counts were determined by looking within the genomic coordinates of each gene and 2,500 bp upstream to include the promoter region. For DNA methylation, genes were increased if there were more hypomethylated DMCs in one cell type versus another. RNA and protein were increased if there was a greater than twofold difference between one cell type versus another. In all cases, neutral refers to no difference being observed between cell types. Using these binary features, a multinomial logistic regression was fitted to each data type, with the reference level being the neutral in all data types. All visualizations were generated in the R statistical environment (v.3.1.3 or higher) using the lattice (v.0.20-31), latticeExtra (v.0.6-26), and BPG (v.5.3.4) packages. For pathway analysis of gene classes with specific relationship states, the g:Profiler web server was used (Reimand et al., 2016), and total overlapping genes (*n* = 3,424) were used as a reference background. Pathways were excluded that contained fewer than three or more than 500 genes or had a query/term intersection of fewer than two genes.

### IF analyses

For IF staining of paraffin-embedded tissue sections, tissue was fixed overnight at 4°C in 4% PFA dissolved in PBS, incubated for a further 24 h at 4°C in 70% ethanol, embedded in paraffin, and sectioned. Tissue sections were deparaffinized in xylene and gradually rehydrated in descending ethanol concentrations. Sections were then treated in Reveal (pH 6.0; Biocare Medical) or Borg (pH 9.5; Biocare Medical) Decloaker RTU antigen retrieval solution for 30 min at 121°C and 10 s at 90°C using a pressure cooker. Tissue was blocked for 1 h at room temperature (20% goat serum, 4% BSA) and incubated with primary antibodies overnight at 4°C. Sections were then washed three times in PBS, incubated with secondary antibodies for 1 h at room temperature, washed two times in PBS and once in distilled water, and mounted using ProLong Gold Antifade (Thermo Fisher Scientific). Staining of frozen mammary tissue sections was performed as described previously (Van Keymeulen et al., 2011). All IF images are 3D composites generated from z-stacks taken on a Zeiss LSM700 confocal microscope using a Plan-Apochromat 40×/1.4-NA oil immersion objective lens. Photos were collected at room temperature, using the following fluorochromes: Alexa Fluor 488 (Thermo Fisher Scientific); indocarbocyanine, Cy3 (Jackson ImmunoResearch); and Alexa Fluor 488 (Thermo Fisher Scientific). Images were collected using the LSM Zen 2012 acquisition software and are representative of three or more biological replicates. ImageJ (National Institutes of Health) was used for 3D reconstitutions.

Antibodies used for IF staining were the same as those listed for intracellular flow cytometry. Additional antibodies used include rabbit anti-DNMT1 (Clone H-300; Santa Cruz); rabbit anti-DNMT3a (ab2850; Abcam); rabbit anti-DNMT3b (ab122932; Abcam); mouse anti-ENMT1 (GLP; clone B0422; R&D Systems); rabbit anti-SET7/SET9 (SETD7; 2813; Cell Signaling); chicken anti-K14 (clone Poly9060; BioLegend); and rat anti-Ki67 (clone SolA15; eBioscience). For IF staining, anti-H3K27me3, anti-EZH2, anti-EHMT2, anti-CREBBP, anti-DNMT3a, anti-DNMT3b, and anti-SET7/SET9 were used on frozen sections. Anti-DNMT1, anti-HDAC1, anti-HDAC2, and anti-EHMT1 were used on paraffin sections. Anti-K14 and anti-Ki67 were used in both staining protocols.

### Whole-mount staining

For whole-mount staining, mammary fat pads were removed, spread onto glass slides, and allowed to adhere at room temperature. Tissue was fixed in Carnoy’s solution (60% ethanol, 30% chloroform, and 10% acetic acid) overnight. Tissue was hydrated with 75%, 50%, and 25% ethanol for 15 min, washed in distilled water, and stained in carmine alum solution (0.2% carmine and 0.5% aluminum potassium sulfate) overnight. Tissue was dehydrated with 70%, 95%, and 100% ethanol for 15 min, cleared in xylene, and mounted using Permount. For side-branching measurements of stained whole mounts, primary, secondary, and tertiary side branches were quantified using a Merz Counting Reticle.

### Quantification and statistical analysis

Details pertaining to the statistical analysis of global proteome, transcriptome, and epigenome data can be found in the relevant Methods sections detailing bioinformatics analyses. For in vivo LDAs, MRU frequencies for LDA experiments were calculated using ELDA software and a 95% confidence interval (Hu and Smyth, 2009). Colony sizes were automatically measured using ImageJ.

For all other experiments, details pertaining to statistical testing methods, biological *n* numbers, and error bars can be found in the relevant figure legends. Unless otherwise indicated, all statistical analyses described below were performed using GraphPad Prism software. For FACS profiling of defined mammary subsets and progenitor enumeration in E- versus EP-treated mice, statistical significance was calculated using two-tailed *t* test or two-way ANOVA and Sidak’s multiple comparisons test. For intracellular flow cytometry analysis, statistical significance was calculated using two-tailed *t* test or two-way ANOVA and Tukey’s multiple comparisons test. For in vitro mouse clonogenic assays, statistical significance for all drug testing comparisons was calculated using two-way ANOVA and Dunnett’s multiple comparisons test. For in vitro human clonogenic assays, statistical significance for all drug testing comparisons was calculated using two-way ANOVA and Dunnett’s multiple comparisons test. Statistical significance for comparison of human breast colony types in wild-type, BRCA1, and BRCA2 patients was calculated using two-way ANOVA and Tukey’s multiple comparisons test.

For short-term in vivo drug treatments and mammopoiesis assays, statistical significance was calculated using one-way (absolute CFC, dead cell frequencies) or two-way (absolute ER^−^PR^−^ basal, ER^−^PR^−^ luminal progenitor, ER^+^PR^+^ luminal) ANOVA followed by Dunnett’s multiple comparisons test. For early- and late-stage apoptotic cell analyses, statistical significance was calculated using two-way ANOVA followed by Sidak’s multiple comparison test. For short-term DAC treatment with and without progesterone, statistical significance was calculated using one-way (absolute CFC) or two-way (absolute ER^−^PR^−^ basal, ER^−^PR^−^ luminal progenitor, ER^+^PR^+^ luminal) ANOVA followed by Tukey’s multiple comparisons test. For prolonged in vivo DAC treatment over 26 wk, statistical significance was calculated using unpaired *t* test (absolute CFC or PROCR^hi^ basal cells) or two-way ANOVA followed by Sidak’s multiple comparisons test.

For in vivo LDAs, MRU frequencies for LDA experiments were calculated using ELDA software and a 95% confidence interval (Hu and Smyth, 2009). For associated data, statistical significance was calculated using unpaired, two-tailed *t* test (absolute CFC, MRU, or PROCR^hi^ basal cells). For Fucci model cell cycle comparisons, statistical significance was calculated using two-way ANOVA followed by Dunnett’s multiple comparisons test. For comparison on tumor initiation in K5-*Cre*;*Trp53*^F/F^ mice treated with PBS versus DAC, statistical significance was calculated using unpaired, two-tailed *t* test.

For *t* tests, ns, P > 0.05; *, P ≤ 0.05; **, P ≤ 0.01; ***, P ≤ 0.001; and ****, P < 0.0001. All multiple comparisons testing, after one- or two-way ANOVAs, were performed with a 0.05 significance level and 95% confidence interval. Statistically significant differences are indicated by asterisks, which denote size of significance levels.

### Data and software availability

The MS data associated with this manuscript have been submitted to a public repository (the Mass Spectrometry Interactive Virtual Environment; http://massive.ucsd.edu). These data are associated with the identifier MSV000079330 at FTP download site: ftp://MSV000079330@massive.ucsd.edu. The microarray data discussed in this study are published (Shiah et al., 2015) and available at National Center for Biotechnology Information (NCBI) Gene Expression Omnibus (Edgar et al., 2002) under accession no. GSE59558. The methylome and ATAC-seq data discussed in this publication have been deposited in NCBI Gene Expression Omnibus and are accessible through accession no. GSE80181.

### Online supplemental material

Figs. S1 and S2 provide additional information and greater context regarding the epigenomes, transcriptomes, and proteomes of primary mammary epithelial cells. Figs. S3, S4, and S5 provide insight into differential expression of key epigenetic master regulators across mammary lineages and the response of adult basal and luminal progenitor cells to epigenetic drugs. Tables S1, S2, S3, and S4 provide extensive data regarding the epigenomes of basal and luminal mammary epithelial cells, as well as how primary epigenomes interact with matching transcriptomes and proteomes. Tables S5 and S7 provide processed proteomics data for mammary epithelial cells, and Tables S6, S8, and S9 give details of GSEA pathway analysis exploring protein changes that occur across mammary lineages and in response to sex hormones.

## Supplementary Material

Supplemental Materials (PDF)

Tables S1-S9 (zipped Excel files)
