# Supplementary material for: Mammary molecular portraits reveal lineage-specific features and progenitor cell vulnerabilities
Source: J Cell Biol. 2018 Aug 6;217(8):2951–74. doi: 10.1083/jcb.201804042 (PMC6080920; doi:10.1083/jcb.201804042)
Supplement: Supplemental Materials (PDF) [file JCB_201804042_sm.pdf]

Supplemental material

Casey et al., https://doi.org/10.1083/jcb.201804042

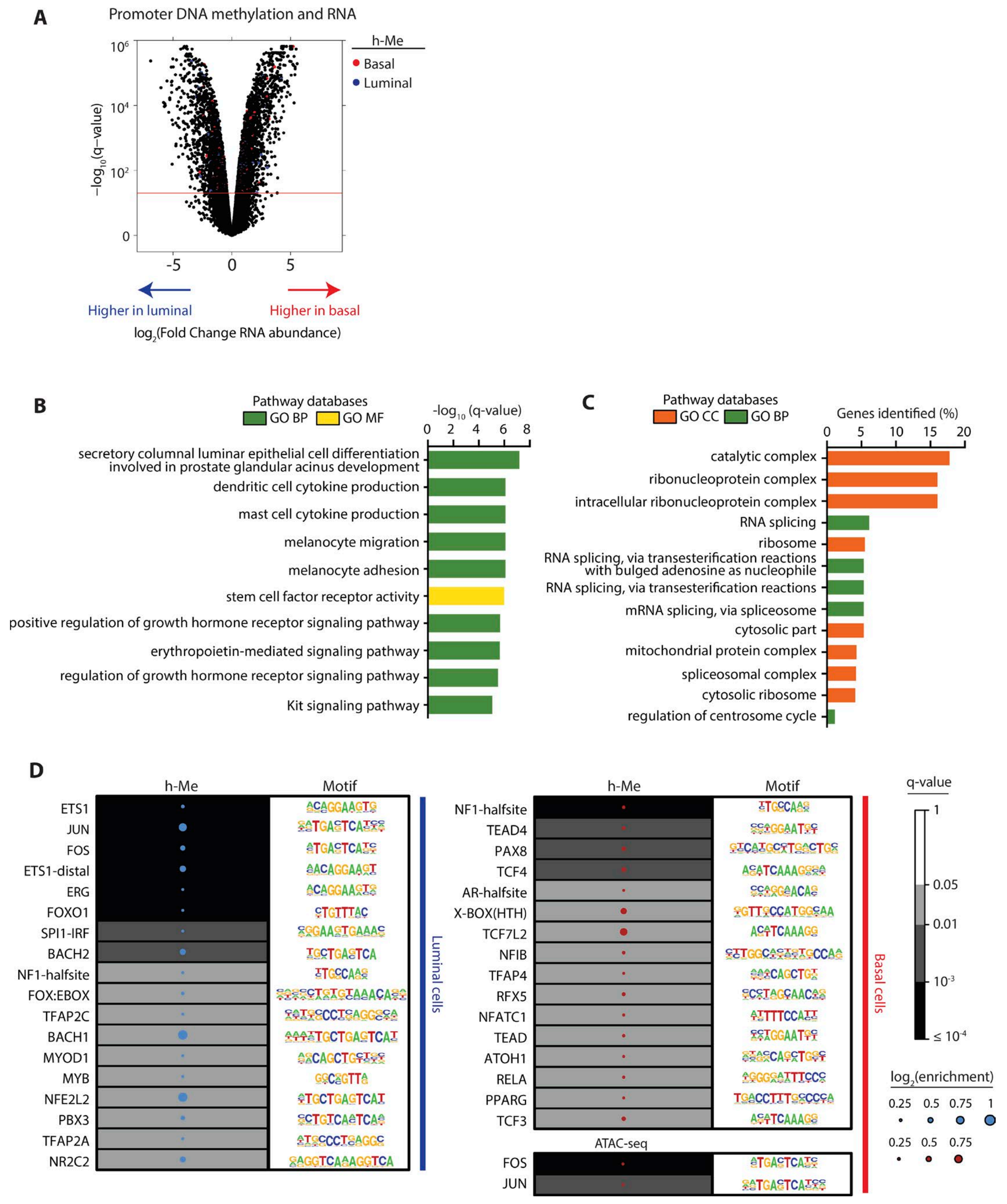

Figure S1. **Building basal and luminal mammary molecular portraits. (A)** Volcano plot shows $\log_2$(FC RNA abundance) across mammary cell compartments; color coding shows RNAs associated with DNA hypomethylation at promoter regions. **(B)** Bar chart shows GO biological pathways (GO BP) and molecular functions (GO MF) found to be enriched in luminal-restricted, open chromatin regions using the GREAT tool. **(C)** Bar chart shows GO cellular component (GO CC) and GO BP enriched for common genes exhibiting no differences in their epigenomes, transcriptomes, or proteomes across mammary lineages using the g:Profiler tool. **(D)** Dot maps show $\log_2$(enrichment over background) and *q*-values for TFBS that are hypomethylated or enriched in open chromatin regions in basal or luminal cells. Tables show HOMER motif logos.

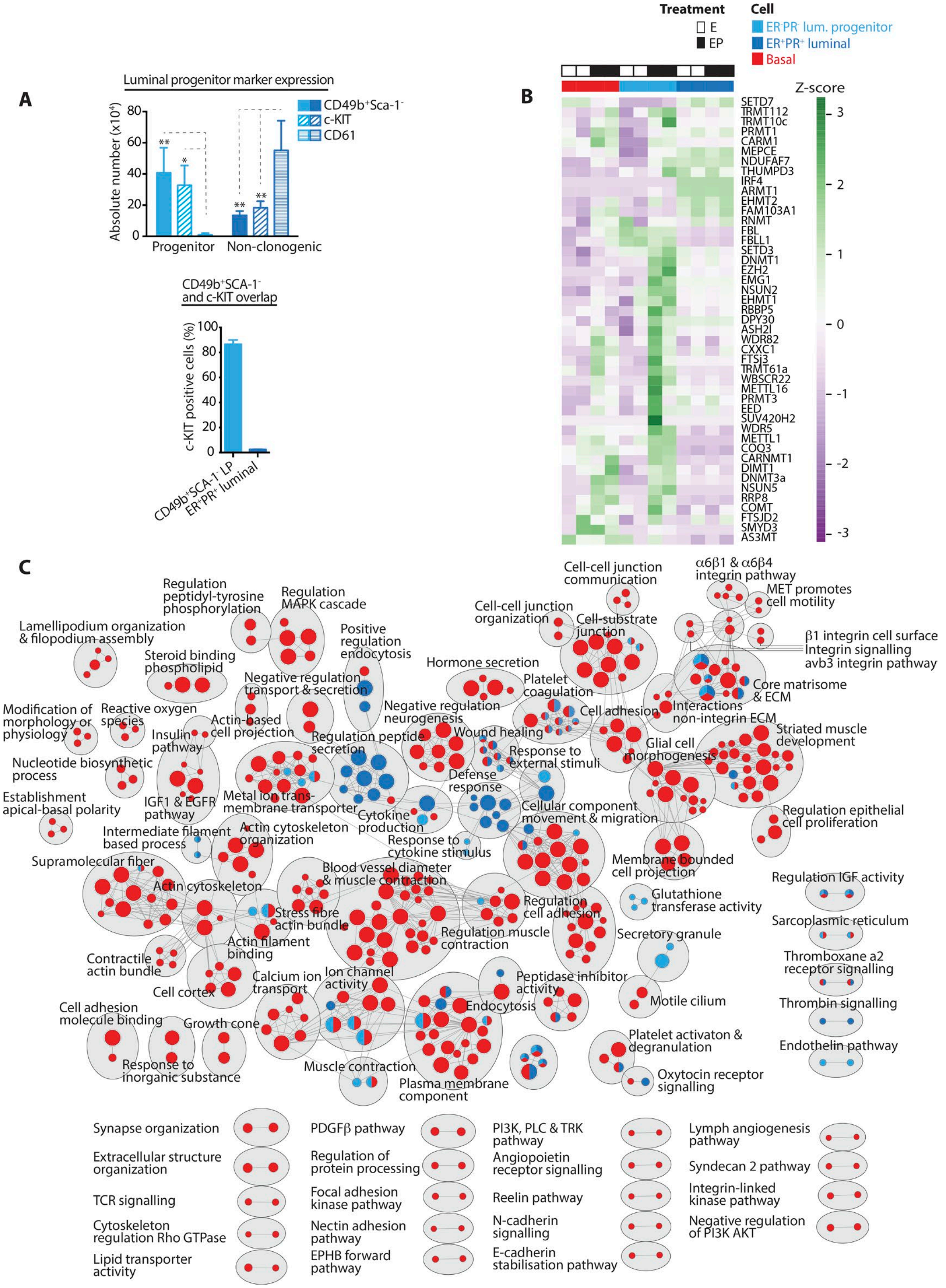

Figure S2. **Identifying distinctions in basal, luminal progenitor, and ER⁺PR⁺ luminal proteomes. (A)** Bar charts show absolute number or relative frequency of luminal cells positive for the indicated cell-surface markers. All absolute quantification was performed per two inguinal mammary glands; biological replicates, *n* = 3; error bars represent SD. Statistical significance was calculated using two-way ANOVA and Sidak's multiple comparisons test performed with a 0.05 significance level and 95% confidence interval. Statistically significant differences are indicated by asterisks, which denote size of significance levels. **(B)** Heatmap shows z-scores of protein abundance for genes associated with SAMe-dependent methyltransferase activity, across samples (biological replicates, *n* = 2). **(C)** Enrichment map visualizes results of GSEA for proteins down-regulated in EP compared with E proteomes. Nodes represent biological pathways that were automatically annotated and organized into themes using Cytoscape; biological themes are labeled and depicted via gray outlines. Colors of nodes show which cell types were enriched for specific pathways (FDR ≤0.05), with multicolored nodes depicting pathways down-regulated by progesterone in two or more subpopulations: ER⁻PR⁻ basal (red), ER⁻PR⁻ luminal progenitor (light blue), and ER⁺PR⁺ luminal (darker blue) cells. Node size is proportional to the number of associated genes.

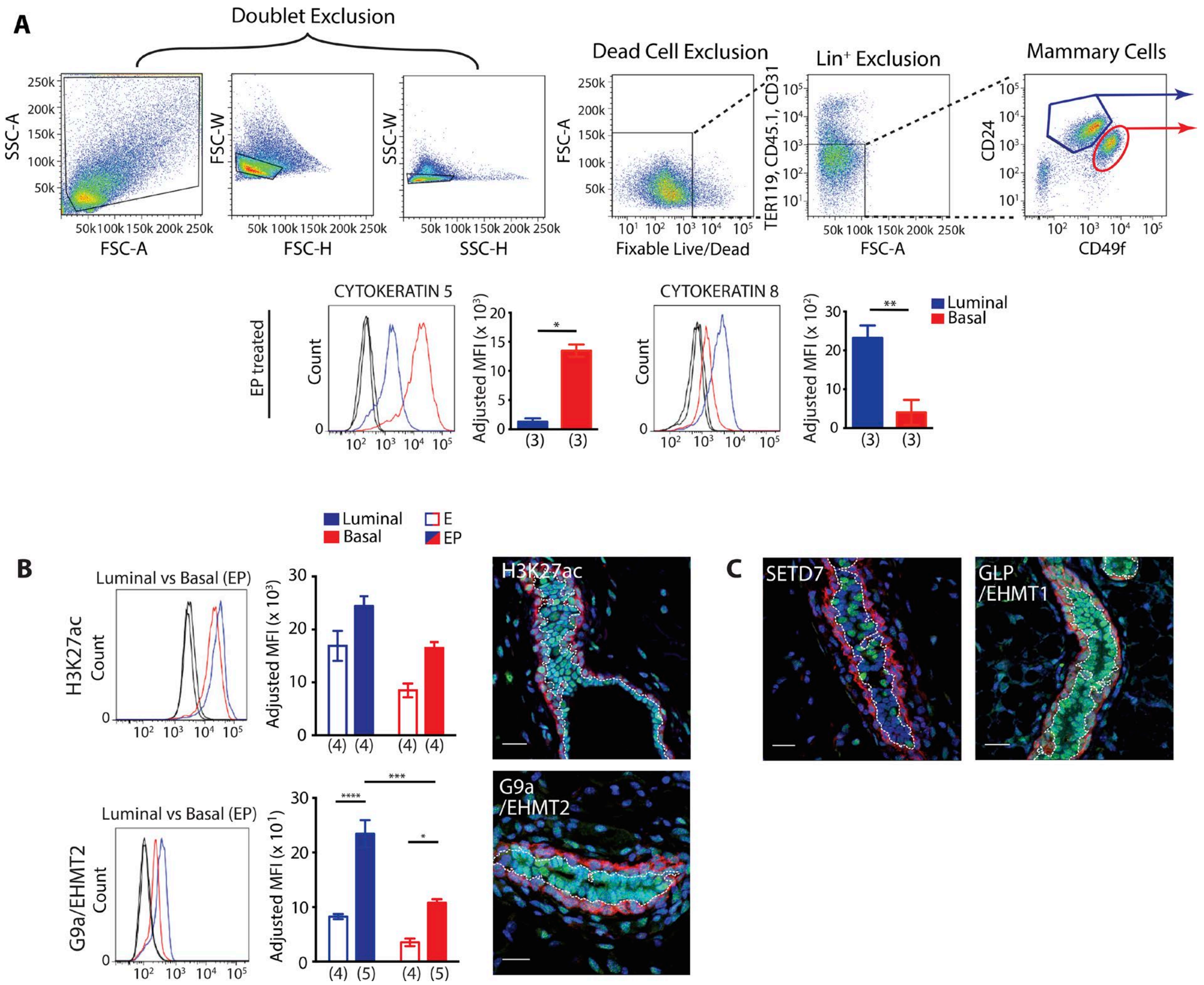

Figure S3. **Spatial and single-cell validation of epigenetic target proteins in the adult mammary gland. (A)** Gating strategy for intracellular flow cytometry of target protein expression in basal and luminal cells. Example histograms show intracellular staining of KRT5 or KRT8 compared with relevant isotype Fc controls. Bar charts show adjusted MFI for each mammary population. Number of biological replicates is shown in brackets under graphs. All error bars represent SEM. Statistical significance was calculated using two-tailed *t* test (*, P ≤ 0.05; **, ≤ 0.01). **(B)** Intracellular flow cytometry of epigenetic targets in mammary epithelial cells. Example histograms show staining for proteins compared with isotype Fc controls (black) in basal (red) and luminal (blue) cells on log scale. Bar charts show adjusted MFI for each mammary population. Number of biological replicates is shown in brackets under graphs. All error bars represent SEM. Statistical significance was calculated using two-way ANOVA and Tukey's multiple comparisons test performed with a 0.05 significance level and 95% confidence interval. Statistically significant differences are indicated by asterisks, which denote size of significance levels. **(B and C)** IF staining of mammary ductal structures in EP-treated mice: DAPI (blue), basal lineage marker K14 (red), and the indicated epigenetic marks or proteins (green). Luminal/basal border is depicted by white dotted line. Bars, 20 µm. *, P ≤ 0.05; ***, P ≤ 0.001; ****, P < 0.0001.

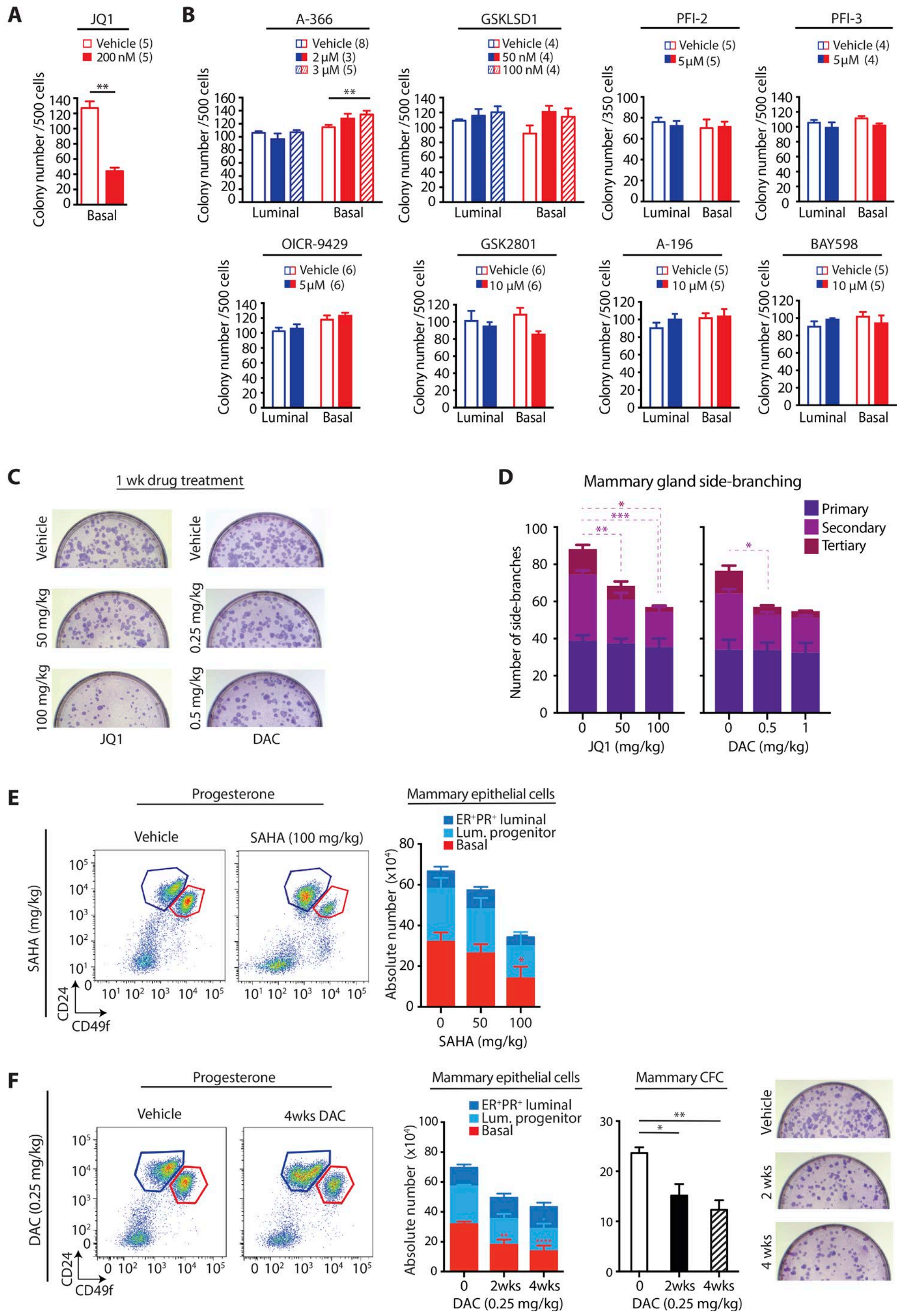

Figure S4. **Pharmacology-mediated inhibition of hormone-induced mammopoiesis in vivo. (A and B)** Bar charts show number of basal or luminal colonies formed with either vehicle control or the indicated concentrations of epigenetic inhibitor. Number of biological replicates is shown in brackets; error bars represent SEM. Statistical significance was calculated using two-tailed *t* test (A) or two-way ANOVA and Sidak's multiple comparisons test (B). **(C)** Representative photographs of colony assay plates from mice treated with progesterone + either vehicle or the indicated concentrations of JQ1 or DAC. **(D)** Bar charts show types of side-branching present in whole mounts taken from mice treated with JQ1 ($n \geq 7$) or DAC ($n \geq 4$). Error bars represent SEM. Statistical significance was calculated using two-way ANOVA and Tukey's multiple comparisons test. **(E and F)** Panels show flow cytometry analysis of luminal ($CD24^{+}CD49f^{lo}$) and basal ($CD24^{-}CD49f^{hi}$) mammary subsets. Left: Stacked bar charts show absolute number of $ER^{-}PR^{-}$ basal, $ER^{-}PR^{-}$ luminal progenitor, and $ER^{+}PR^{+}$ luminal cells, which were further purified using the CD49b and SCA-1 cell-surface markers. Primary mammary cells were purified from the two inguinal glands; error bars represent SEM. Statistical significance was calculated using one-way (absolute CFC) or two-way ANOVA (absolute $ER^{-}PR^{-}$ basal, $ER^{-}PR^{-}$ luminal progenitor, $ER^{+}PR^{+}$ luminal) followed by Dunnett's multiple comparisons test. **(E)** Mice were treated with vehicle or 100 mg/kg SAHA daily for 1 wk, + progesterone. Biological replicates, $n \geq 5$; error bars represent SEM. **(F)** Mice were treated with 0.25 mg/kg DAC, five weekly doses for 4 wk. Right: Bar charts shows absolute number of total mammary CFC. Biological replicates, $n \geq 3$; error bars represent SEM. Photographs show representative colony assay plates for total mammary cells. For all panels, multiple comparisons testing was performed with a 0.05 significance level and 95% confidence interval; statistically significant differences are indicated by asterisks, which denote size of significance levels. *, $P \leq 0.05$; **, $P \leq 0.01$; ***, $P \leq 0.001$.

https://doi.org/10.1083/jcb.201804042

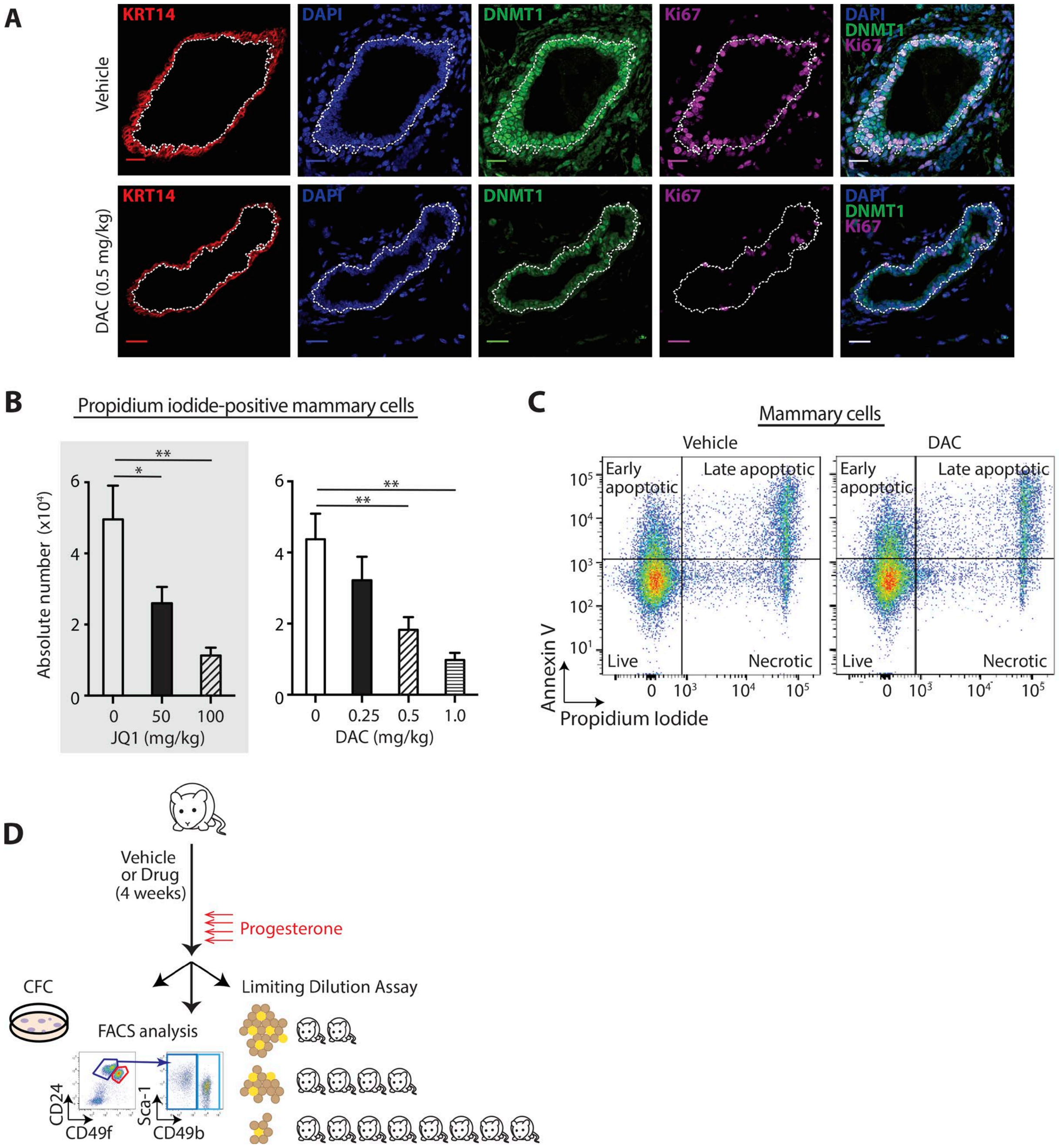

Figure S5. **JQ1 and DAC exert a cytostatic effect on the mammary epithelium and prevent progesterone-driven mitogenic responses. (A)** IF staining of mammary ductal structures in mice treated with progesterone + either vehicle or 0.5 mg/kg DAC, daily for 1 wk. DAPI (blue), Ki67 (magenta), basal lineage marker K14 (red), and DNMT1 (green). Luminal/basal border is depicted by white dotted line. Bars, 20 µm. **(B)** Bar charts show absolute number of primary mammary dead cells purified from the two inguinal glands of mice treated for 1 wk with JQ1 (gray background) or DAC treatment, + progesterone. Dead cells were determined via propidium iodide staining; biological replicates, *n* ≥7 or *n* ≥ 4, respectively. Statistical significance was calculated using one-way ANOVA followed by Dunnett's multiple comparisons testing performed with a 0.05 significance level and 95% confidence interval. Statistically significant differences are indicated by asterisks, which denote size of significance levels. **(C)** Representative FACS plots of early- and late-apoptotic mammary cells after treatment with 0.5 mg/kg DAC, three weekly doses for 4 wk. Apoptotic cells were measured via staining of lin^- mammary cells with annexin V and propidium iodide. Biological replicates, *n* = 5. **(D)** Workflow schematic for LDA experiments. *, $P \leq 0.05$; **, $P \leq 0.01$.

Provided online are nine tables in Excel.

Table S1 shows GREAT pathway analysis for lineage-restricted ATAC-seq peaks in basal and luminal cells.

Table S2 shows statistics from multinomial logistic regression models fitted for each data type (open chromatin, DNA methylation, RNA abundance, and protein abundance) in basal and luminal cells.

Table S3 shows genes proximal to basal cell hypomethylated TFBS and open chromatin regions.

Table S4 shows genes proximal to luminal cell hypomethylated TFBS and open chromatin regions.

Table S5 shows basal and luminal mammary proteomes.

Table S6 shows GSEA results for proteins up-regulated in basal versus luminal proteomes.

Table S7 shows ER^-PR^- basal, ER^-PR^- luminal progenitor, and ER^+PR^+ luminal proteomes generated from E- versus EP-treated mice.

Table S8 shows GSEA results for proteins up-regulated in ER^-PR^- basal, ER^-PR^- luminal progenitor, and ER^+PR^+ luminal cells by progesterone.

Table S9 shows GSEA results for proteins down-regulated in ER^-PR^- basal, ER^-PR^- luminal progenitor, and ER^+PR^+ luminal cells by progesterone.
